# *S*‑Oxide *peri*-Annulated Blatter Radicals: A Paradigm for Chiral Radicals

**DOI:** 10.1021/jacs.6c10282

**Published:** 2026-06-30

**Authors:** Paulina Bartos, Emilia Obijalska, Anna Pietrzak, Piotr Kaszyński

**Affiliations:** † Faculty of Chemistry, 49602 University of Łódź, Tamka 12, 91-403 Łódź, Poland; ‡ Faculty of Chemistry, Łódź University of Technology, Żeromskiego 114, 90-543 Łódź, Poland; § Centre of Molecular and Macromolecular Studies, Polish Academy of Sciences, Sienkiewicza 112, 90-363 Łódź, Poland

## Abstract

Selective oxidation of the sulfur atom in two S-*peri*-annulated Blatter radicals with Oxone gives racemic sulfoxides, one of which was resolved into enantiomers and characterized by electronic circular dichroism (ECD). They are the first examples of a potentially broad class of centrally chiral radicals, in which the endocyclic chirality center in the π polycyclic radical skeleton represents a new paradigm in the structural chemistry of radicals. A higher ratio of Oxone gives analogous endocyclic sulfones. The resulting stable radicals were characterized by spectroscopic (UV–vis, ECD, EPR), electrochemical, XRD, and DFT methods.

## Graphical abstract

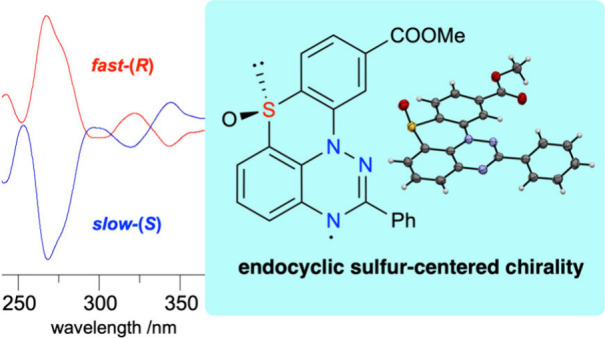

Chiral organic radicals are of fundamental interest and increasing significance in emerging technologies based on spin-dependent phenomena,^1^ such as molecular electronics,^2^ spintronics^3–5^ (including chirality-induced spin selectivity,^6–8^ CISS), and quantum computing.^9^ Such radicals with spin delocalized in the π system directly affected by the chiral field are still rare.^10,11^ A handful of known, electrically neutral chiral radicals are either helicenes^12–20^ or derived from planarly prochiral [2,2]­paracyclophane.^21,22^ Unfortunately, many of them have limited stability.

A class of particularly robust radicals consists of the π-delocalized Blatter radical^23^ and its derivatives,^24^ which are based on the benzo­[*e*]­[1,2,4]­triazinyl (BT) core. They exhibit favorable and tunable electrochemical, magnetic, and electronic properties.^25,26^ For these reasons they have been intensely investigated in the context of modern materials,^27^ including high-spin systems,^28–31^ liquid crystals,^32–35^ and NIR absorbers,^36,37^ intended for energy storage,^38–40^ molecular electronics,^41,42^ sensors,^43,44^ and spintronics.^9,45–48^ The robustness, rich chemistry and chemical versatility of the spin bearing BT unit make it well suitable for functionalization and incorporation into more complex molecular architectures. In this context, we recently demonstrated fusion of the BT unit with helicenes (**A** ^49^ and **B**,^50^ Figure 1), as well as planarly (**C**)^51^ and axially chiral (**D** ^52^ and **E**)^53^ open-shell derivatives.

**Figure 1:**
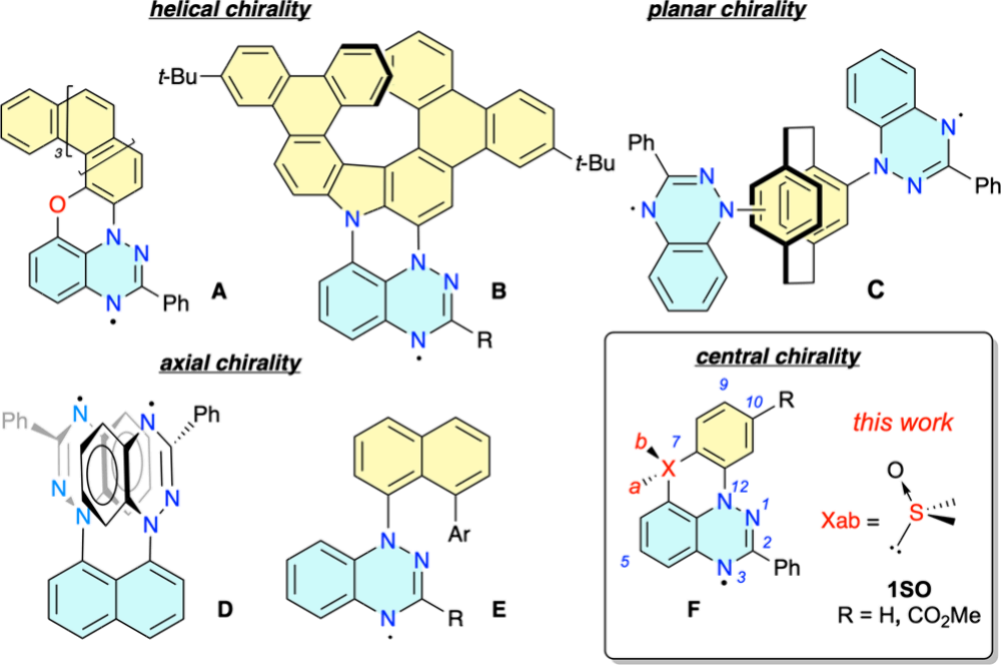
Four classes of chiral derivatives of the benzo­[*e*]­[1,2,4]­triazinyl: helically chiral **A** (ref 49) and **B** (ref 50), planarly chiral **C** (ref 51), axially chiral **D** (ref 52) and **E** (ref 53), and centrally chiral **F**. In **B** and **E** R = Ph, *t*-Bu. The partial numbering system for **F** is shown.

*peri*-Annulation of the Blatter radical, leading to planar Blatter radicals,^54–58^ permits incorporation of a configurationally stable, tetrahedral atom X (as the divalent >X*ab* group) into the ring system, leading to radicals with the chiral core of the general structure **F** (*a* ≠ *b*, Figure 1). This divalent endocyclic group can, in principle, be carbon (as >C*R*_1_ *R*_2_), nitrogen (as >N^+^ *R*_1_ *R*_2_), or phosphorus (e.g., >P­(O)*R* and >P­(O)*OR*) based. While these groups introduce a configurationally stable chirality center to the planar Blatter core, they do not participate in the π system. More promising in this context is the sulfoxyl group, >S=O, containing an electron pair capable, in principle, of weak π interactions.

Herein, we report the first centrally chiral Blatter radicals **1SO** (Figure 1), in which the stereogenic sulfur atom is part of the π ring system. We describe an efficient and chemoselective synthesis of two sulfoxides, the parent **1SO-a** and its functionalized analogue **1SO-b** (Figure 1). The sulfoxides and side product sulfones **1SO**_**2**_ are characterized by structural (**1SO-b**), spectroscopic, and electrochemical methods. The effect of the S-center oxidation on electronic properties and spin delocalization is assessed. The racemate of **1SO-b** is resolved into enantiomers, and ECD spectra are recorded. All experimental results are augmented with DFT computations.

The racemic sulfoxides **1SO** were prepared by controlled, chemoselective oxidation of sulfur-annulated planar Blatter radicals^54,57^ **1S** (Scheme 1). Initial screening of oxidants revealed that although DMDO^59,60^ and *m*CPBA^61^ rapidly oxidized the sulfur center, they led to poor selectivity and uncontrolled overoxidation to sulfones **1SO**_**2**_. In contrast, oxidation with Oxone (KHSO_5_)^62^ under buffered, biphasic conditions provided superior selectivity. Thus, treatment of sulfide **1S** with 1.5 equiv of Oxone in the presence of NaHCO_3_ in a CH_2_Cl_2_/acetone/H_2_O mixture at 0–5 °C resulted in the clean conversion of **1S** to **1SO** within 3–5 min. Under these conditions, **1SO-a** and **1SO-b** were obtained as the major products in good, isolated yields (52–57% and 65–73%, respectively), with only trace amounts of the corresponding sulfones **1SO**_**2**_ (4–9%). Good yields of the sulfones, up to 73%, were obtained using 2.5 equiv of Oxone and extending the reaction time to 10 min.

**Scheme 1:**
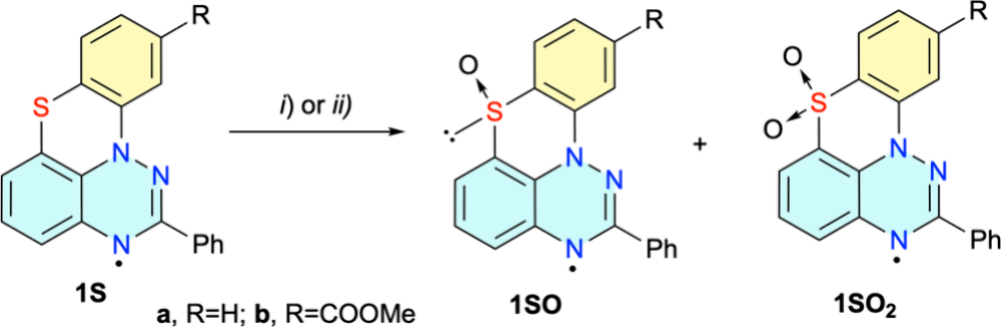
Synthesis of Radicals **1SO** and **1SO**_**2**_ ^a^

a Reagents and conditions: *i*) Oxone 1.5 equiv, aq. NaHCO_3_, CH_2_Cl_2_/acetone, 0–5 °C, 3-5 min: **1SO-a** 52–57% and **1SO**_**2**_ **-a** 4–9%, **1SO-b** 65–73% and **1SO**_**2**_ **-b** 4-6%. *ii*) Oxone 2.5 equiv, aq. NaHCO_3_, CH_2_Cl_2_/acetone, 0–5 °C, 10 min: **1SO**_**2**_ **-a** 59–73%, **1SO**_**2**_ **-b** 70%.

The molecular structure of **1SO-b** was confirmed by single crystal XRD methods. Analysis revealed an approximately planar heterocyclic structure with a small, 8° puckering along the N(12)···S(7) line, 1.490(3) Å for the S(7)–O distance, and 109.9(1)° for the N(12)···S(7)–O angle (Figure 2). The first two values are smaller while the angle is larger than those typical for phenothiazine *S*-oxides (average 29°, 1.50 Å, and 93°, respectively).^63–68^

**Figure 2:**
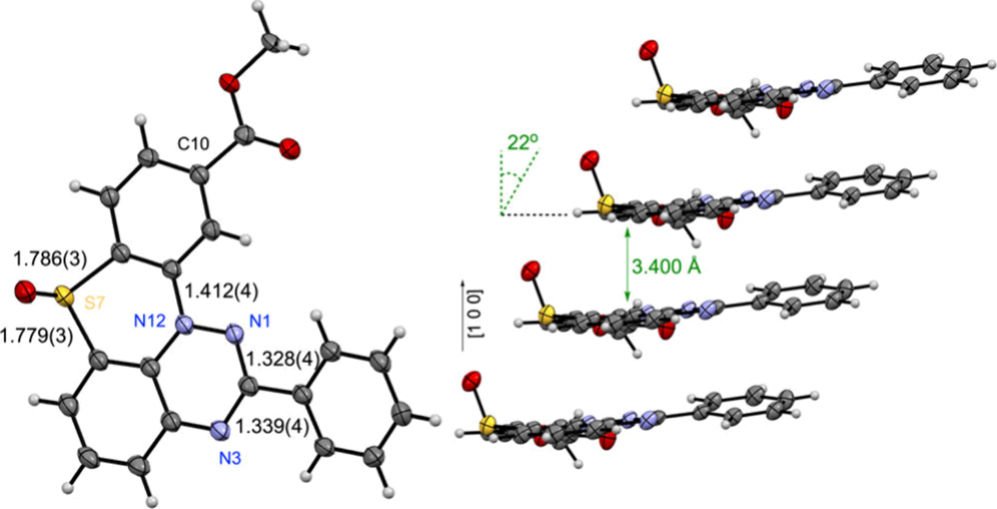
Left: Atomic displacement ellipsoid diagram for radical **1SO-b** with partial labeling and pertinent dimensions (ellipsoids set at 50% probability). Right: Partial packing diagram for **1SO-b**. For full details, see the SI.

Molecules of **1SO-b** form homochiral slipped stacks with an interplanar separation of 3.400 Å and a slippage angle of 22° (Figure 2). The stacks are arranged in sheets parallel to the ***ac*** plane with alternating polar (S=O) and nonpolar (Ph) interaction regions separated by 11.73 and 13.03 Å, respectively. The sheets are separated by 10.81 Å and connected through interactions of the COOMe groups. DFT calculations for a pair of neighboring molecules within the stack revealed moderate antiferromagnetic interactions with *J*/*k*_B_ = −28 K. For comparison, **1S-a** exhibits weak intermolecular ferromagnetic interactions in the solid state (*J*/*k*_B_ = +10.3 K),^54^ while the typically observed antiferromagnetic interactions in Blatter radical derivatives can be over an order of magnitude stronger.^69^

Electronic absorption spectroscopy demonstrated that oxidation of the sulfur center in **1S** results in a significant hypsochromic shift of the absorption bands in the spectra of sulfoxides **1SO** and sulfones **1SO**_**2**_, which now resemble the parent Blatter radical^70^ (Figure 3). TD-DFT calculations indicate that the lowest-energy absorption bands in **1SO** and **1SO**_**2**_ arise from the closely spaced D_0_ → D_1_ and D_0_ → D_2_ electronic excitations, both of which are dominated by the α-HOMO → α-LUMO and β-HOMO → β-LUMO transitions. In comparison to **1S**, oxidation of the S-center nearly completely removes the sulfur atom contribution to the Frontier Molecular Orbitals (FMOs), which results in the observed hypsochromic shift (Figure 3).

**Figure 3:**
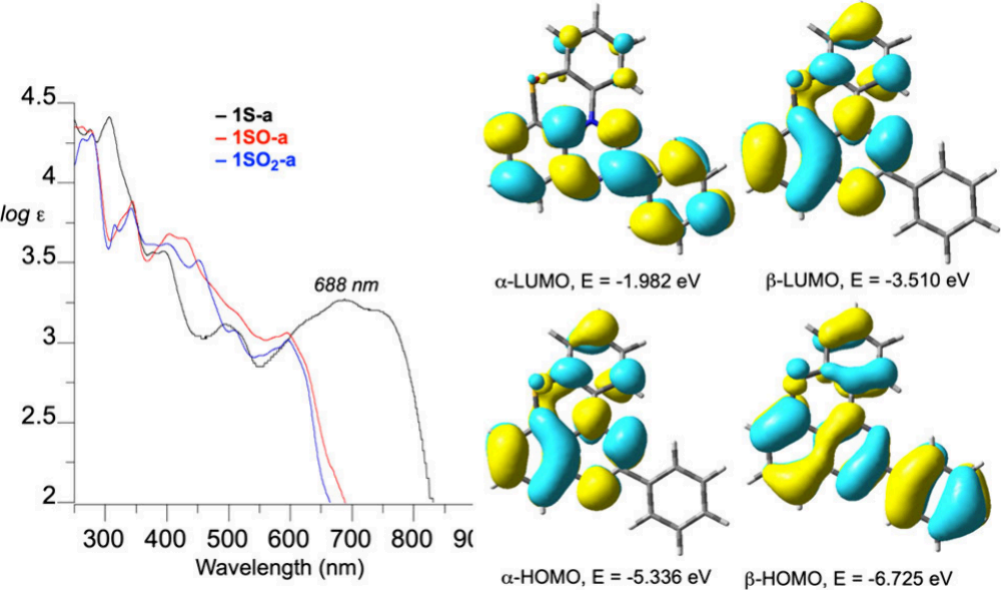
Left: Electronic absorption spectra of **1S-a**, **1SO-a**, and **1SO**_**2**_ **-a** in CH_2_Cl_2_. Right: UB3LYP/6-311+G­(2d,p)//UB3LYP/6-311G­(2d,p)-derived MO contours and energies relevant to low-energy excitations of **1SO-a** in CH_2_Cl_2_ dielectric medium (MO isovalue = 0.02).

The racemic sulfoxide **1SO-b** was resolved by chiral semipreparative HPLC, and electronic circular dichroism (ECD) spectra were recorded for both enantiomers (Figure 4). The absolute configurations of the individual enantiomers were assigned as *S* for the first-eluted enantiomer (*fast*-**1SO-b**) and *R* for the second enantiomer (*slow*-**1SO-b**) by comparison of the experimental ECD spectra with those calculated using TD-DFT (see the Supporting Information (SI) for details).

**Figure 4:**
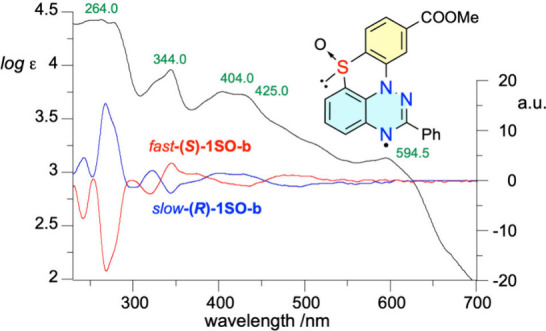
Electronic absorption spectrum of **1SO-b** (left axis) and qualitative ECD spectra of enantiomers of **1SO-b** in CH_2_Cl_2_. Assignment based on comparison with TD-DFT results. *Fast* and *slow* refer to the order of HPLC elution.

The configurational stability of the sulfoxide stereocenter in **1SO** was examined using DFT methods. Thus, the racemization barrier (Δ*H* ^‡^) for **1SO-a** was calculated in vacuum as 36.6 kcal mol^–1^, which is in good agreement with the experimental value of Δ*H* ^‡^ = 36.2 kcal mol^–1^ reported^71^ for tolyl phenyl sulfoxide in xylene (DFT calculated Δ*H* ^‡^ = 38.6 kcal mol^–1^ in vacuum). These results indicate the high configurational stability of sulfoxides **1SO** below 100 °C, sufficient for handling and chemical manipulations.

Cyclic voltammetry (CV) measurements reveal quasi-reversible redox processes for radicals **1SO** and **1SO**_**2**_ (Figure 5). As summarized in Table 1, stepwise oxidation of the sulfur atom in the planar Blatter scaffold induces anodic shifts of the redox potentials, by approximately 0.35 V for **1SO-a** and 0.46 V for **1SO**_**2**_ **-a** relative to **1S-a**. These systematic shifts reflect a progressive stabilization of both HOMO and LUMO energy levels upon oxidation of the sulfur center (Figure 5), consistent with the increasing electron-withdrawing character of the sulfoxide and sulfone functionalities. Similar trends are found for series **b** with the COOMe substituent (Table 1).

**Figure 5:**
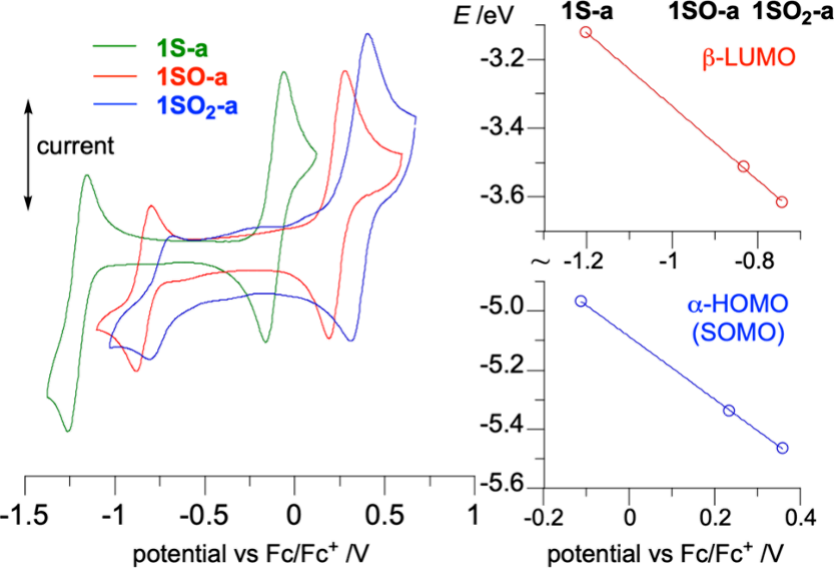
Left: Cyclic voltammograms for **1S-a**, **1SO-a**, and **1SO**_**2**_ **-a** referenced to the Fc/Fc^+^ couple. Conditions: 0.5 mM CH_2_Cl_2_ in [*n*-Bu_4_N]^+^[PF_6_]^−^ (50 mM) at *ca.* 20 °C, 50 mV s^–1^, scans from 0 V in the anodic direction, glassy carbon working electrode, Pt counter electrode, and Ag/AgCl pseudoreference electrode. Right: Redox potentials vs FMOs energies for radicals (UB3LYP/6-311+G­(2d,p)//UB3LYP/6-311G­(2d,p) method in CH_2_Cl_2_ dielectric medium).

**Table 1:** Electrochemical,^a^ EPR,^b^ and DFT^c^ Data for Radicals **1**

Radical	*E*_1/2_^–1/0^/V	*E*_1/2_^0/+1^/V	*a*_N12_/G^d^	*a*_H6_/G^d^	ρ_S7_ × 10^3^ ^e^	RDV^–1^ ^f^
**1S-a** ^g^	–1.202	–0.112	7.54	1.18	30.5	3.618
**1SO-a**	–0.833	0.232	4.47	2.96	–1.6	3.340
**1SO_2_-a**	–0.745	0.358	4.49	3.34	5.7	3.232
**1S-b** ^g^	–1.103	–0.047	7.08	1.78	31.1	3.615
**1SO-b**	–0.782	0.280	4.95	3.10	3.0	3.305
**1SO_2_-b**	–0.756	0.361	4.24	3.89	5.6	3.205

a Potential vs the Fc/Fc^+^ couple. See Figure 5 for details.

b Recorded in benzene at *ca.* 20 °C.

c UCAM-B3LYP/EPR-III//UB3LYP/6-311G­(2d,p) level of theory in the benzene dielectric medium.

d The *hfcc* value is ascribed to the N(12) or H­(C6) atom in **1** on the basis of DFT results.

e Spin density on the S(7) group (S, SO, or SO_2_).

f RDV = Σ­(ρ_i_)^2^. See the SI for details.

g Ref 57.

Detailed analysis of the experimental and computational results for **1SO** and **1SO**_**2**_ demonstrates good correlations of the redox potentials with Hammett σ_p_ parameters^72^ for model substituents −SPh, −SOPh, and −SO_2_Ph (Figure 6).

**Figure 6:**
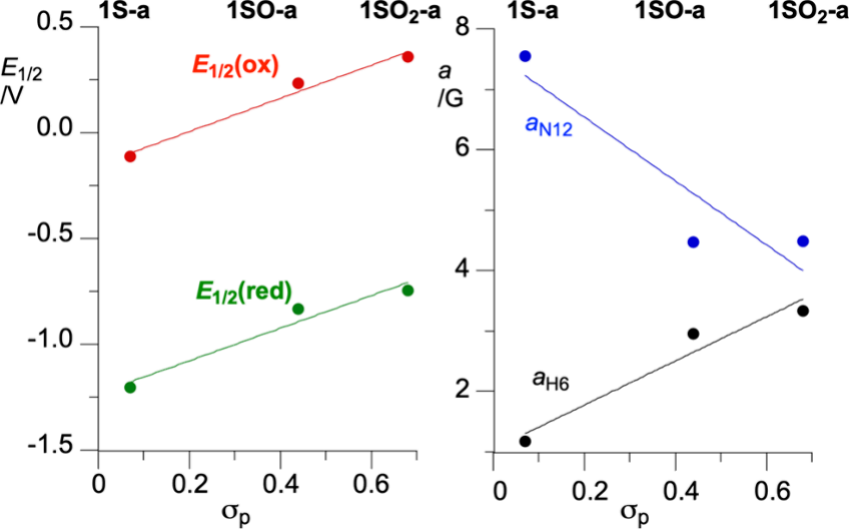
Left: Correlation of *E*_1/2_^0/+1^ (red) and *E*_1/2_^–1/0^ (green) with Hammett σ_p_ parameters. Right: Correlation of *a*_N12_ (blue) and *a*_H6_ (black) *hfcc* with Hammett σ_p_ parameters for model groups −SPh, −SOPh, and −SO_2_Ph. See the SI for details.

Finally, EPR spectroscopy shows that oxidation of the S-center in **1S** significantly alters the spin distribution in the radicals (Figure 7 and Table 1). Thus, the *a*_N12_ *hfcc* values systematically decrease, while those for *a*_H6_ *hfcc* increase upon stepwise oxidation **1S** → **1SO** → **1SO**_**2**_ in both series of compounds (R = H and COOMe, Table 1 and Figure 6). These trends are paralleled by the decreasing overall spin delocalization (decreasing RDV^–1^ value) and markedly diminished spin concentration at the sulfur atom (ρ_S7_, Table 1). The latter is consistent with marginal interactions of the S atom lone pair with the π system in **1SO**, which is corroborated by the observed negligible participation in the FMOs (UV–vis results) and a relatively high barrier to epimerization calculated for **1SO-a**.

**Figure 7:**
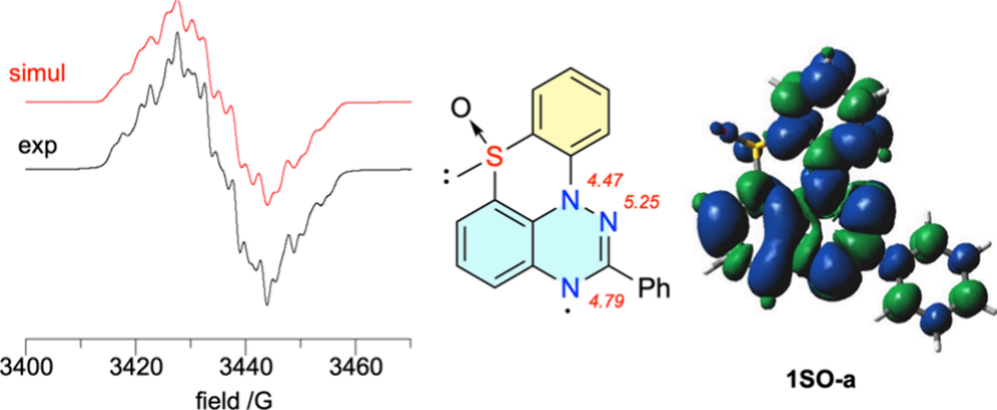
Left: Experimental (black) and simulated (red) EPR spectra for **1SO-a** recorded in benzene at *ca.* 20 °C. Right: Assignment of the resulting *hfcc* (G) and the total spin density map in **1SO-a** based on DFT calculations in benzene dielectric medium. Contours are plotted at ± 0.02 (e/bohr^3^)^1/2^.

In conclusion, we have demonstrated the fourth type of chirality in highly persistent benzo­[*e*]­[1,2,4]­triazinyl radicals, which represents a new structural paradigm in chiral radicals. Thus, the previously reported Blatter derivatives with axial, planar, and helical chirality are now complemented with centrally chiral derivatives **1SO** containing the endocyclic sulfoxide group. In all four types of radicals the π-delocalized electron spin is part of the chiral field generated by the structure or by an endocyclic stereogenic center. The presently reported two sulfoxides **1SO** constitute the first examples of a potentially broad class of centrally chiral radicals of the general structure **F**.

The chiral sulfoxides **1SO** are obtained by simple, efficient, and chemoselective oxidation of radicals **1S**. The straightforward synthesis, high persistence, and presence of a functional group in **1SO-b** open up opportunities for the development of chiral paramagnetic building blocks for use in advanced materials with spin-dependent phenomena.

## Supplementary Material



## Data Availability

All computational results are deposited in the Zenodo repository at 10.5281/zenodo.19729818.

## Notes

Full synthetic and characterization details; XRD collection and refinement information; UV–vis, ECD, and EPR spectra; and electrochemical and computational results (PDF)

## Notes

On the occasion of the 85th birthday of Professor Fred Wudl.
