# Supplementary material for: S‑Oxide peri-Annulated Blatter Radicals: A Paradigm for Chiral Radicals
Source: J Am Chem Soc. 2026 Jun 30;148(27):28055–60. doi: 10.1021/jacs.6c10282 (PMC13383730; doi:10.1021/jacs.6c10282)
Supplement: Supplementary file 1 [file ja6c10282_si_001.pdf]

Supporting Information
for

S-Oxide *peri*-Annulated Blatter Radicals: A Paradigm for Chiral Radicals

Paulina Bartos,[a] Emilia Obijalska,[a] Anna Pietrzak,[b] and Piotr Kaszyński* [a,c]

[a] Faculty of Chemistry, University of Łódź, Tamka 12, 91-403 Łódź, Poland.
[b] Faculty of Chemistry, Łódź University of Technology, Żeromskiego 114, 90-543 Łódź, Poland
[c] Center of Molecular and Macromolecular Studies, Polish Academy of Sciences, Sienkiewicza 112, 90-363 Łódź, Poland
* Email: piotr.kaszynski@chemia.uni.lodz.pl

Table of Content:

1. Synthetic details S2
2. XRD data collection and refinement S4
3. Electronic absorption spectroscopy S7
4. Chiral HPLC analysis and resolution S10
5. Electronic circular dichroism spectroscopy S12
6. Electrochemical results S13
7. EPR spectra S16
8. Computational details and results S18
a) isotropic Fermi contact coupling constants (hfcc) S18
b) spin delocalization of radicals in benzene dielectric medium S20
c) magnetic exchange interactions in the crystal structure S22
d) electronic excitations S22
e) partial output data from TD-DFT calculations for radicals S25
f) determination of absolute configuration of enantiomers S30
g) DFT calculation of sulfoxides racemization S31
9. Archive for UB3LYP/6-311G(2d,p) geometry optimization results for **1** S32
10. References S37

1. Synthetic details

General. Reagents and solvents were obtained from commercial suppliers. For separation of radicals high-purity grade silica gel (w/ "Ca", ~0.1%; purchased from Sigma-Aldrich, product no. 60752-1KG) were used. IR spectra were recorded using an Agilent Cary 630 FTIR spectrometer, in neat. UV spectra were measured in CH_2Cl_2 on PerkinElmer Lambda 45 spectrophotometer. Melting points were determined on a Stuart SMP30 Advanced Digital Melting Point Apparatus and are uncorrected. High-resolution mass spectrometry (HRMS) measurements were performed using SYNAPT G2-Si High Resolution Mass Spectrometry equipped with an ESI or APCI source and Quantitative Time-of-Flight (QuanTof) mass analyzer.

Preparation of radicals 1S

Radicals **1S-a** and **1S-b** were synthesized according to the previously published procedure.[1]

Preparation of racemic radicals 1SO (procedure A)

Radical **1S** (0.0637 mmol) was dissolved in a CH_2Cl_2/acetone mixture (v/v = 2:1, 2–3 mL) in a round-bottom flask and cooled to 0–5 °C using an ice bath. To the vigorously stirred solution were added saturated aqueous $NaHCO_3$ (1 mL) followed by Oxone® (30 mg, 0.0977 mmol, 1.5 equiv) dissolved in a minimal amount of water. The reaction progress was monitored by TLC (CH_2Cl_2) at short time intervals. The reaction was quenched upon complete consumption of the starting radical **1S** (green–gray spot; $R_{f\,\mathbf{1S\text{-}a}}$ = 0.80, $R_{f\,\mathbf{1S\text{-}b}}$ = 0.66), typically after **3–5 min**. Careful monitoring was required, as the initially formed sulfoxide undergoes rapid over-oxidation to the sulfone under prolonged reaction times. Upon completion, water (~5 mL) and CH_2Cl_2 (~5 mL) were added, and the layers were separated. The aqueous phase was extracted with CH_2Cl_2 (2×5 mL). The combined organic layers were dried (Na_2SO_4), filtered, and concentrated under reduced pressure. The crude product was purified by column chromatography on SiO_2 (w/ "Ca", ~0.1%), eluting with CH_2Cl_2 containing increasing amounts of ethyl acetate (0–50%). The desired sulfoxide radical **1SO** was isolated as the major product (brown spot; CH_2Cl_2/EtOAc 9:1, $R_{f\,\mathbf{1SO\text{-}a}}$ = 0.46, $R_{f\,\mathbf{1SO\text{-}b}}$ = 0.44), accompanied by minor amounts of the corresponding sulfone **$1SO_2$**. For details see Table S1.

Preparation of radicals $1SO_2$ (procedure B)

Radical **1S** (0.0637 mmol) was dissolved in a CH_2Cl_2/acetone mixture (v/v = 2:1, 2–3 mL) and cooled to 0–5 °C. Saturated aqueous $NaHCO_3$ (1 mL) was added, followed by Oxone® (50 mg, 0.163 mmol, 2.5 equiv) dissolved in a small amount of water. The reaction mixture was stirred vigorously and monitored by **TLC** until complete disappearance of both the starting radical **1S** ($R_{f\,\mathbf{1S\text{-}a}}$ = 0.80, $R_{f\,\mathbf{1S\text{-}b}}$

= 0.66) and the intermediate sulfoxide **1SO** (brown spot; CH_2Cl_2/EtOAc 9:1, $R_{f\,\mathbf{1SO\text{-}a}}$ = 0.46, $R_{f\,\mathbf{1SO\text{-}b}}$ = 0.44), typically after **~10 min**. In cases of incomplete conversion, an additional portion of Oxone® (≈10% of the initial amount) was added. Workup and extraction were performed as described in Procedure A. Purification by column chromatography on SiO_2 (w/ "Ca", ~0.1%), using CH_2Cl_2 with 0–10% ethyl acetate, afforded the desired sulfone radical **$1SO_2$** as the main product (brown spot; CH_2Cl_2, $R_{f\,\mathbf{1SO2\text{-}a}}$ = 0.73, $R_{f\,\mathbf{1SO2\text{-}a}}$ = 0.52). For details see Table S1.

2-Phenyl-3H-[1,2,4]triazino[5,6,1-kl]phenothiazin-3-yl 7-oxide (**1SO-a**)

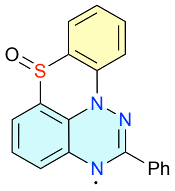

Yield: 52-57% (11-12 mg, procedure A)

Mp. 188–189°C

IR (neat) *v* 2921, 2851, 1456, 1428, 1393, 1271, 1216, 1041, 764, 692 cm^{-1}.

UV-vis (CH_2Cl_2) λ_{max} (log ε) 595.5 (3.06), 424 (3.65), 404.0 (3.68), 344.0 (3.88), 277.0 (4.325), 263.0 (4.35), 252.0 (4.35) nm.

HRMS (TOF AP^+) *m/z* $[M+H]^+$ calcd $C_{19}H_{13}N_3OS$: 331.0779, found: 331.0788.

Anal. Calcd. for $C_{19}H_{12}N_3OS$ (330.39): C, 69.07; H, 3.66; N, 12.72; S, 9.70. Found: C, 69.09; H, 3.70; N, 12.77; S, 9.73.

Methyl 2-phenyl-3H-[1,2,4]triazino[5,6,1-kl]phenothiazin-3-yl-10-carboxylate 7-oxide (**1SO-b**)

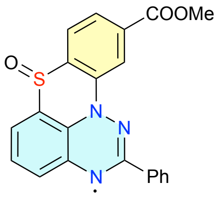

Yield: 65-73% (16-17 mg, procedure A)

Mp. 207–209°C

IR (neat) *v* 1715, 1557, 1413, 1275, 1245, 1033, 969, 913, 760, 683 cm^{-1}.

UV-vis (CH_2Cl_2) λ_{max} (log ε) 594.5 (3.13), 424.5.0 (3.73), 404.0 (3.75), 344.0 (3.96), 277.0 (4.40), 263.0 (4.43), 252.0 (4.42) nm.

HRMS (TOF AP+) *m/z* $[M+H]^+$ calcd $C_{21}H_{15}N_3O_3S$: 389.0834, found: 389.0844.

Anal. Calcd. for $C_{21}H_{14}N_3O_3S$ (388.42): C, 64.94; H, 3.63; N, 10.82; S, 8.25. Found: C, 64.89; H, 3.58; N, 10.84; S, 8.26.

2-Phenyl-3H-[1,2,4]triazino[5,6,1-kl]phenothiazin-3-yl 7,7-dioxide (**$1SO_2$-a**).

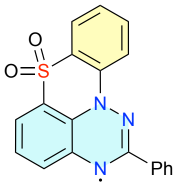

Yield: 4-9% (1-2 mg, procedure A) Yield: 59-73% (13-16 mg, procedure B)

Mp. >250°C (decomp.)

IR (neat) *v* 2922, 2853, 1581, 1469, 1436, 1398, 1290, 1145, 1088, 758, 679 cm^{-1}.

UV-vis (CH_2Cl_2) λ_{max} (log ε) 595.5 (3.015), 509.0 (3.08), 451.5 (3.51), 399.5 (3.62), 342.0 (3.84), 315.5 (3.74), 279.0 (4.31), 263.5 (4.27), 238.0 (4.14) nm.

HRMS (TOF AP^+) *m/z* $[M+H]^+$ calcd $C_{19}H_{13}N_3O_2S$: 347.0728, found: 347.0737.

Anal. Calcd. for $C_{19}H_{12}N_3O_2S$ (346.38): C, 65.88; H, 3.49; N, 12.13; O, 9.24; S, 9.26. Found: C, 65.75; H, 3.25; N, 12.06; S, 9.24.

***Methyl 2-phenyl-3H-[1,2,4]triazino[5,6,1-kl]phenothiazin-3-yl-10-carboxylate 7,7-dioxide* (1SO₂-b).**

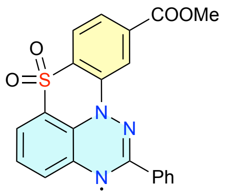

Yield: 4-6% (1-2 mg, procedure A) Yield: 70%(18 mg, procedure B)

Mp. >250°C (decomp.)

IR (neat) *v* 1723, 1422, 1394, 1281, 1143, 1107, 978, 757, 742, 694 cm^{-1}.

UV-vis (CH_2Cl_2) λ_{max} (log ε) 595.0 (3.07), 425.5 (3.73), 401.5 (3.73), 342.5 (3.89), 321.0 (3.86), 276.5 (4.37), 264.0 (4.40), 239.5 (4.40) nm.

HRMS (TOF AP^+) *m/z* $[M+H]^+$ calcd $C_{21}H_{15}N_3O_4S$: 405.0783, found: 405.0787.

Anal. Calcd. for $C_{21}H_{14}N_3O_4S$ (404.42): C, 62.37; H, 3.49; N, 10.39; O, 15.82; S, 7.93. Found: C, 62.35; H, 3.50; N, 10.42; S, 8.02.

Table S1. Summary of oxidation experiments and formation of radicals **1SO** and **1SO₂**.

Radical	R_f		Yield [%] (mg)	
	CH_2Cl_2	CH_2Cl_2:AcOEt (9:1)	*Procedure A*	*Procedure B*
**1S-a**	0.80	1.00	-	-
**1S-b**	0.66	1.00	-	-
**1SO-a**	0.07	0.46	52-57% (11-12 mg)	-
**1SO-b**	-	0.44	65-73% (16-17 mg)	-
**1SO₂-a**	0.73	1.00	4-9% (1-2 mg)	59-73% (13-16 mg)
**1SO₂-b**	0.52	1.00	4-6% (1-2 mg)	70% (18 mg)

2. XRD data collection and refinement for 1SO-b

Single-crystal XRD measurement for **1SO-b** was performed with a Rigaku XtaLAB Synergy, Pilatus 300K diffractometer. The measurement was conducted at 100 (3) K using the CuK_α radiation (λ = 1.54184 Å). The data was integrated using CrysAlisPro program.[2] Intensities for absorption of **1SO-b**

were corrected using multi-scan method. SCALE3 ABSPACK scaling algorithm implemented in CrysAlisPro program has been used.

CCDC: File 2492834 contain the supplementary crystallographic data for this paper. These data can be obtained free of charge from The Cambridge Crystallographic Data Centre via www.ccdc.cam.ac.uk/structures

Structure solution and refinement

The structure was solved with the ShelXT[3] structure solution program using Intrinsic Phasing and refined in the ShelXle by the full-matrix least-squares minimization on F^2 with the ShelXL[4] refinement package. All non-hydrogen atoms were refined anisotropically, and C–H hydrogens were generated geometrically using the HFIX command as in ShelXL. Hydrogen atoms were refined isotropically and constrained to ride on their parent atoms.

The crystal data and structure refinement descriptors are presented in Table S2. The molecular structure and partial packing diagrams are shown in Figures S1–S3.

Table S2. Selected structural data for 1SO-b

	**1SO-b**<br>CCDC: 2492834
Formula	$C_{21}H_{14}N_3O_3S$
Formula Weight	388.42
Crystal System	triclinic
Space Group	$P\bar{1}$
a/Å	4.2716(4)
b/Å	10.8078(5)
c/Å	18.4619(7)
α/°	81.623(4)
β/°	85.465(5)
γ/°	87.143(6)
Volume/Å^3	839.95(9)
Z	2
2θ range for data collection/°	8.276 to 134.138
Index ranges	$-4 \leq h \leq 5$, $-12 \leq k \leq 12$, $-21 \leq l \leq 21$
No. of measured, independent, and observed [$I>2\sigma(I)$] reflections	15708,2956, 2452
R_{int}	0.0558
Goodness-of-fit on F^2	1.085
Final R indexes [$F^2 > 2\sigma(F^2)$]	$R_1 = 0.0599$, $wR_2 = 0.1555$
Final R indexes [all data]	$R_1 = 0.0713$, $wR_2 = 0.1637$
Data/restraints/parameters	2956/0/254
Largest diff. peak/hole Å^{-3}	0.50/-0.59

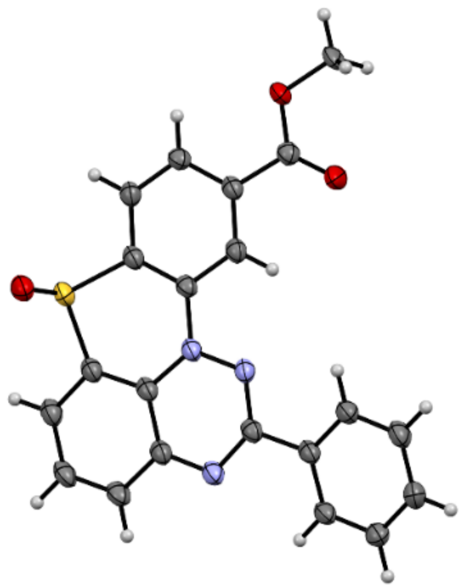

Figure S1. Molecular structure of **1SO-b**. Displacement ellipsoids are drawn at 50% probability level.

Racemic radical **1SO-b** crystallizes in triclinic system in $P\bar{1}$ space group. The crystal system contains one symmetry independent molecule (Figure S1) defined by nearly planar molecular core with angles between mean planes of the benzo[*e*][1,2,4]triazine fragment and the adjacent benzene ring are 6.9° and 11.1°, respectively. The puckering along the N12···S7 line, measured as the angle between mean planes of the adjacent benzene rings, is 8°. Similarly, C2-Ph group in **1SO-b** is almost coplanar with the polycyclic fragment with the angle between mean planes of benzo[*e*][1,2,4]triazine and the phenyl group of 9.8°. Bond lengths S7-C6a (1.779(3) Å) and S7-C7a (1.786(3) Å) in **1SO-b** are elongated. Similarly, in COOMe-derivatives N12-C2a' bond lengths are elongated (1.389(4)Å for **1SO-b**). A supramolecular assembly of the studied structure may be defined as stacks extending along [1 0 0] direction (Figures S2 and S3).

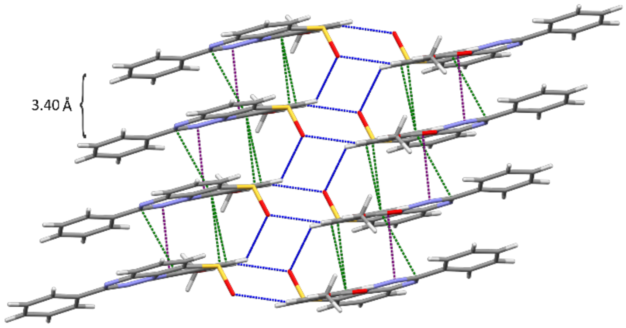

Figure S2. Partial packing diagram of **1SO-b**. Individual interactions are marked: C···C – green; N···O-purple; C-H···O – blue. Depicted contacts are 0.1Å shorter than sum of relevant vdW radii.

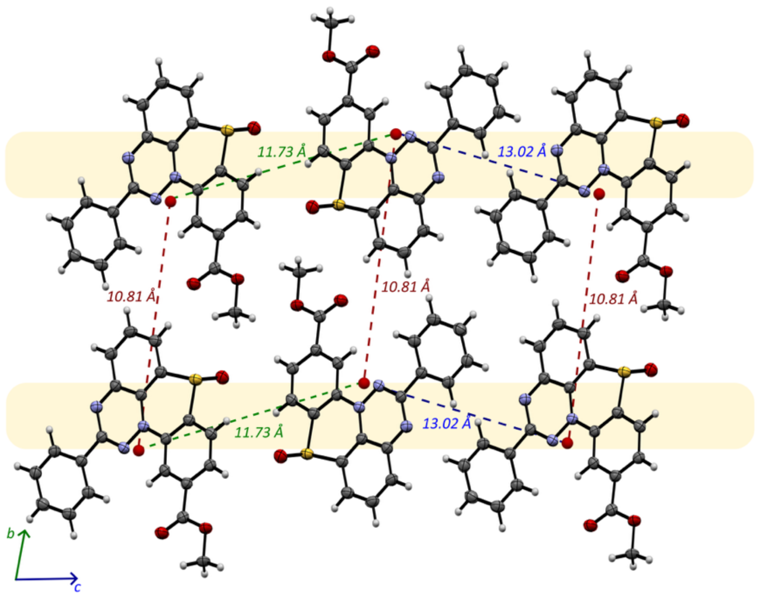

Figure S3. Top view along the *a*-axis of neighboring stacks assembled in parallel sheets (*ac* plane, yellow highlighted), which form the crystal structure of **1SO-b**. The indicated distances correspond to separations between the axes of neighboring stacks and sheets: distance between homochiral stacks separated by a polar region, 11.73 Å (green dashed lines), distance between homochiral stacks separated by a non-polar region, 13.02 Å (blue dashed lines), and distance between the sheets, 10.81(red dashed lines).

Supramolecular stacks of **1SO-b** are stabilized by π···π stacking interactions represented by short (<vdW-0.1 Å) C···C and C···N contacts between aromatic fragments of molecules. Additionally, stacked molecules are associated through C-H···O interactions. Neighboring stacks are linked through C-H···O hydrogen bonds engaging S=O group as an acceptor.

The resulting stacks in **1SO-b** are characterized by interplanar distances between mean planes of benzo[*e*][1,2,4]triazine fragments of 3.40 Å.

3. Electronic absorption spectroscopy

Electronic absorption spectra for radicals **1SO** and **$1SO_2$** were recorded in spectroscopic grade CH_2Cl_2 at concentrations in a range 1.0–5× 10^{-5} M and fitted to the Beer–Lambert law. Results are shown in Figures S4–S7.

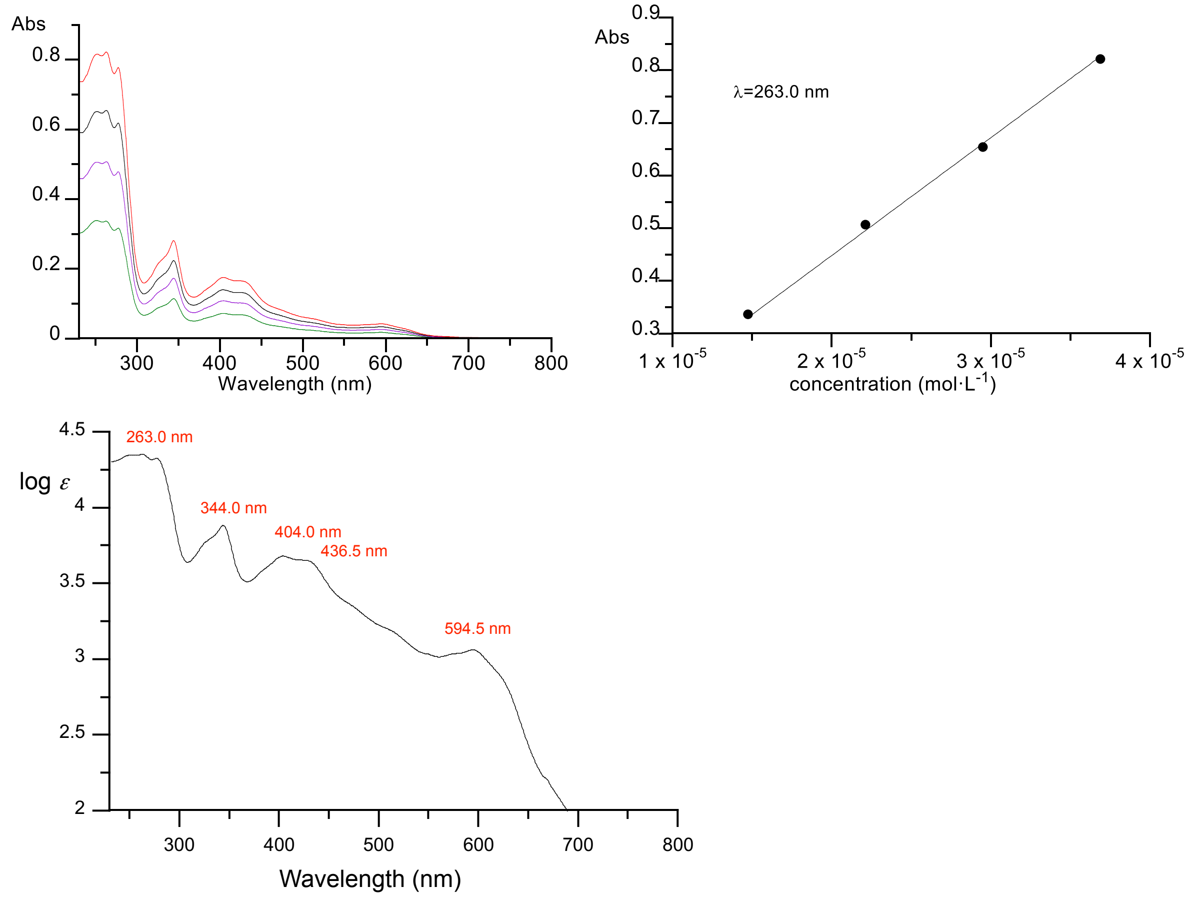

Figure S4. Clockwise: electronic absorption spectra for **1SO-a** in CH_2Cl_2 for four concentrations, determination of molar extinction coefficient ε at λ = 263.0 nm (best fit function: ε = 22400(160)×conc, r^2 = 0.998), molar extinction *log* (ε) plot.

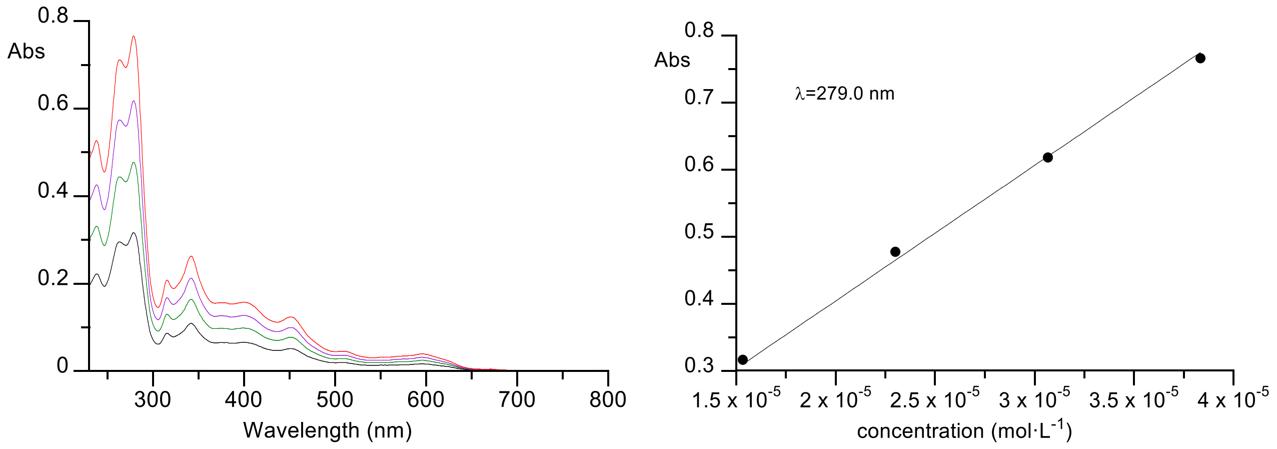

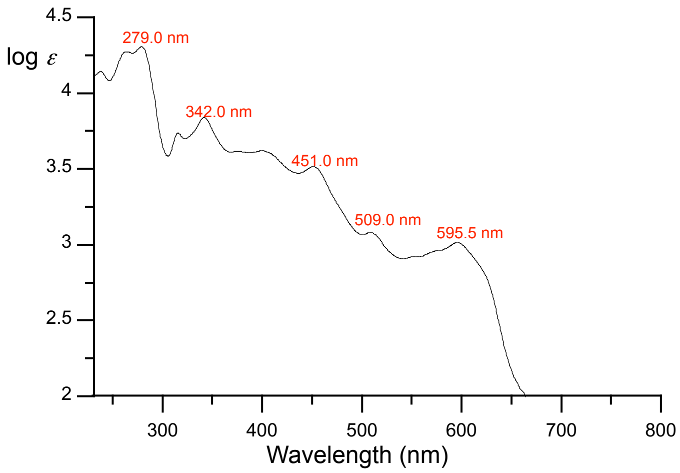

Figure S5. Clockwise: electronic absorption spectra for **1SO$_2$-a** in CH_2Cl_2 for four concentrations, determination of molar extinction coefficient ε at λ = 279.0 nm (best fit function: ε = 20210(171)×conc, r^2 = 0.998), molar extinction *log* (ε) plot.

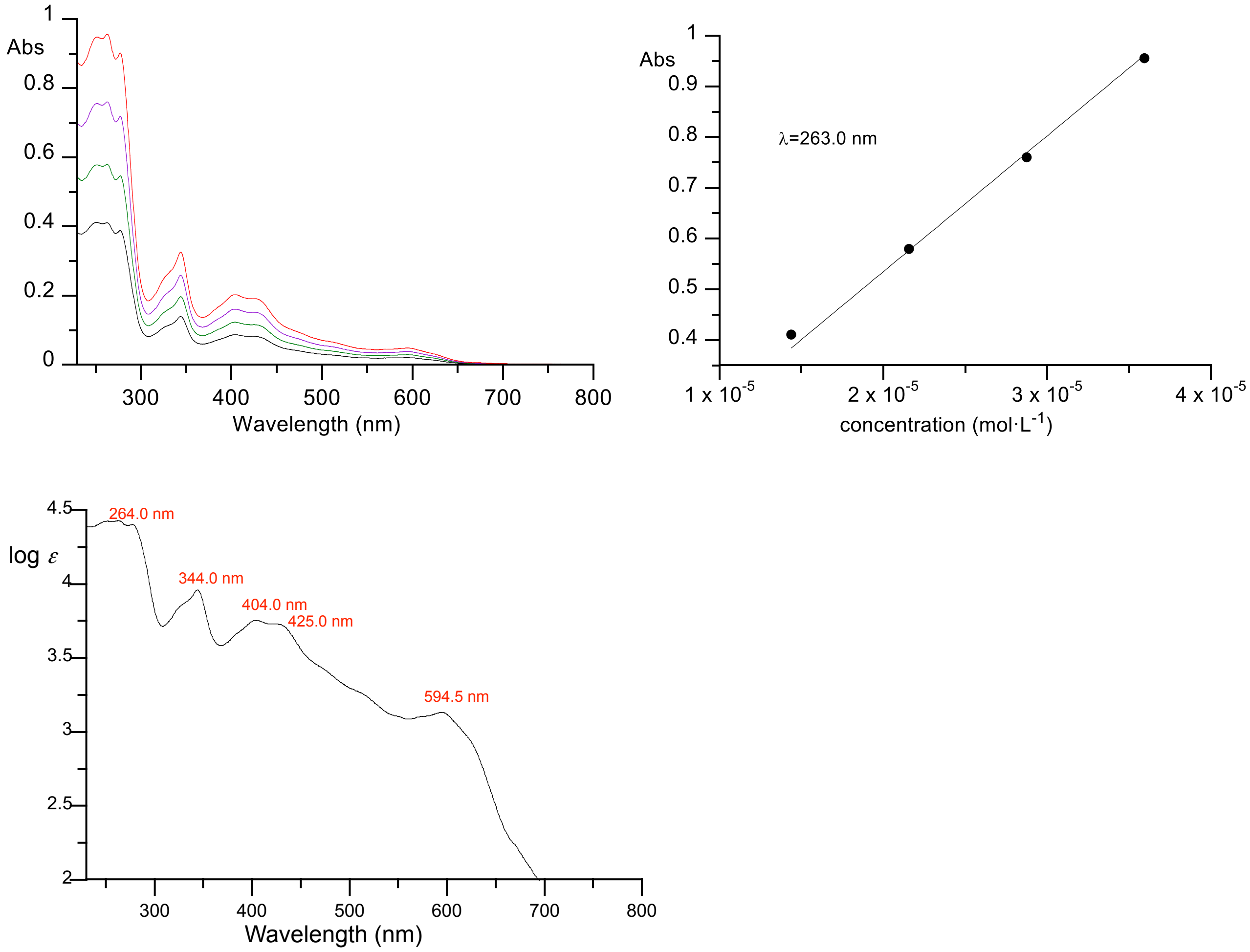

Figure S6. Clockwise: electronic absorption spectra for **1SO-b** in CH_2Cl_2 for four concentrations, determination of molar extinction coefficient ε at λ = 263.0 nm (best fit function: ε = 26746(312)×conc, r^2 = 0.995), molar extinction *log* (ε) plot.

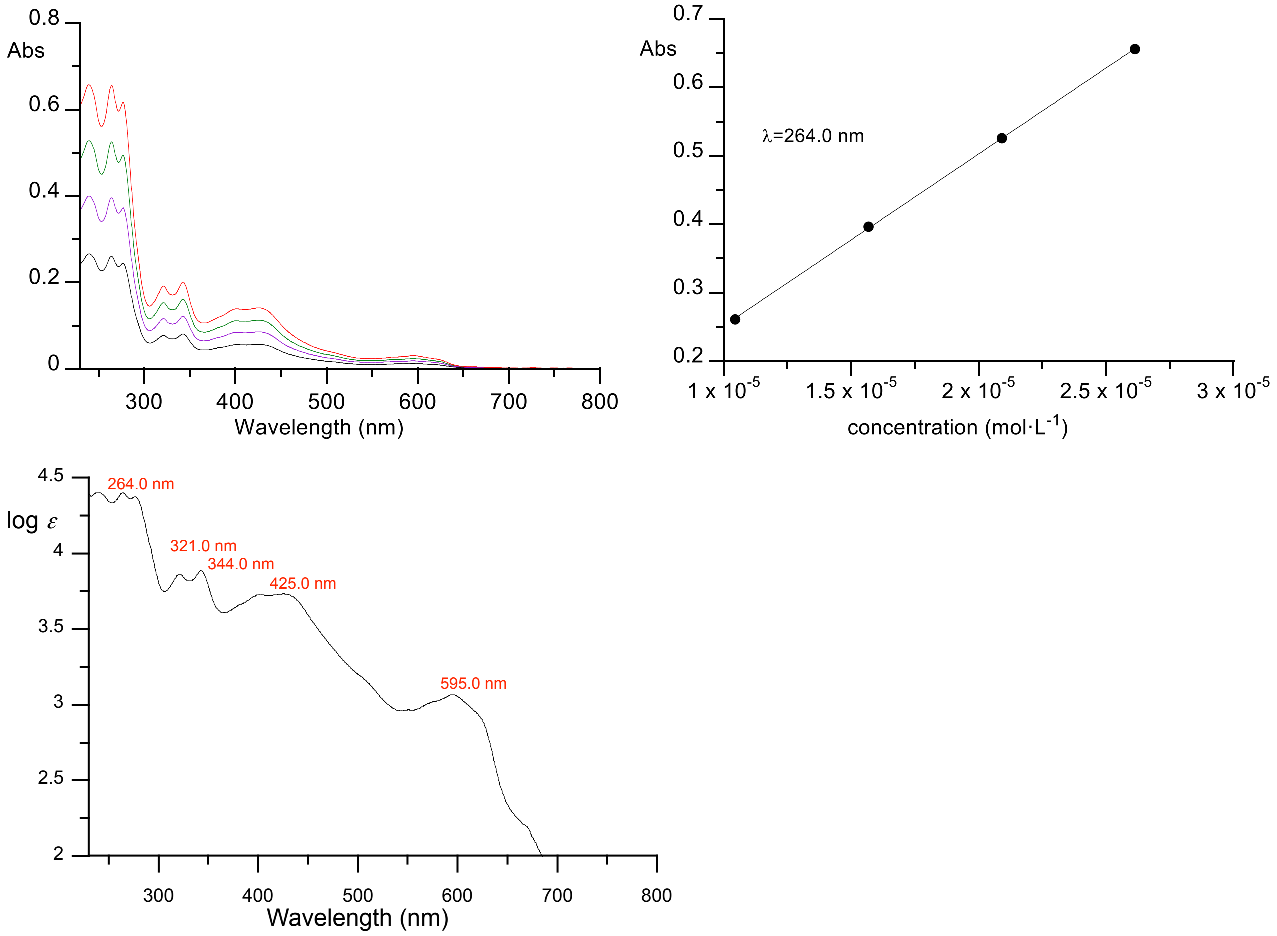

Figure S7. Clockwise: electronic absorption spectra for **1SO$_2$-b** in CH_2Cl_2 for four concentrations, determination of molar extinction coefficient ε at λ = 264.0 nm (best fit function: ε = 25121(47)×conc, r^2 = 0.999), molar extinction *log* (ε) plot.

4. Chiral HPLC analysis and resolution

Chiral HPLC analyses were performed on CHIRALPAK® IC analytical column (cellulose tris-3,5-dimethylphenylcarbamate, 250×4.6 mm) using a CH_2Cl_2/hexane/i-PrOH mixtures (65:30:5 ratio for radical **1SO-a** and 55:30:7 ratio for radicals **1SO-b**, **1SO$_2$-a**, **1SO$_2$-b**) with a flow rate 1.0 mL/min and UV detection 254 nm. Retention times t_R are given in minutes. Chromatograms are shown in Figures S8-S12.

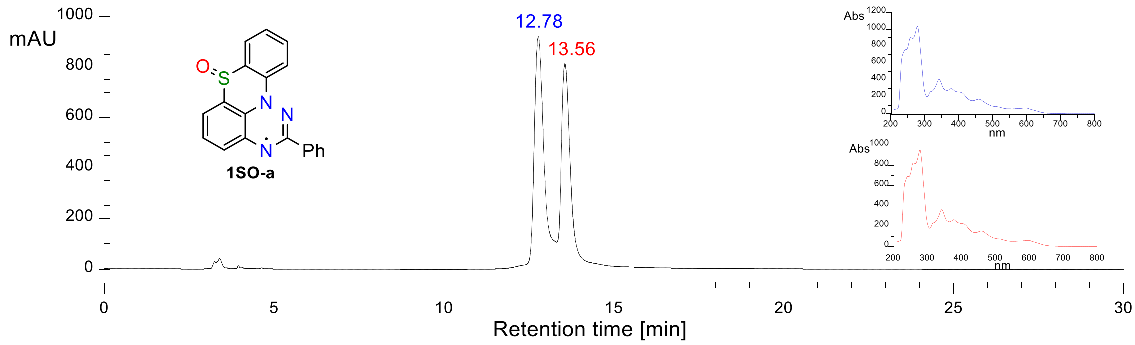

Figure S8. Chiral HPLC analysis of racemic radical **1SO-a** using CHIRALPAK® IC column monitored at 254 nm.

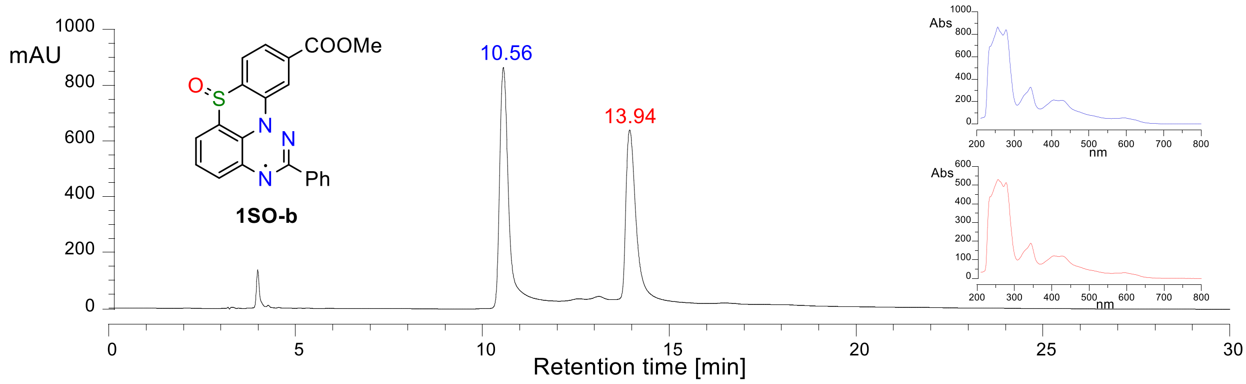

Figure S9. Chiral HPLC analysis of racemic radical **1SO-b** using CHIRALPAK® IC column monitored at 254 nm.

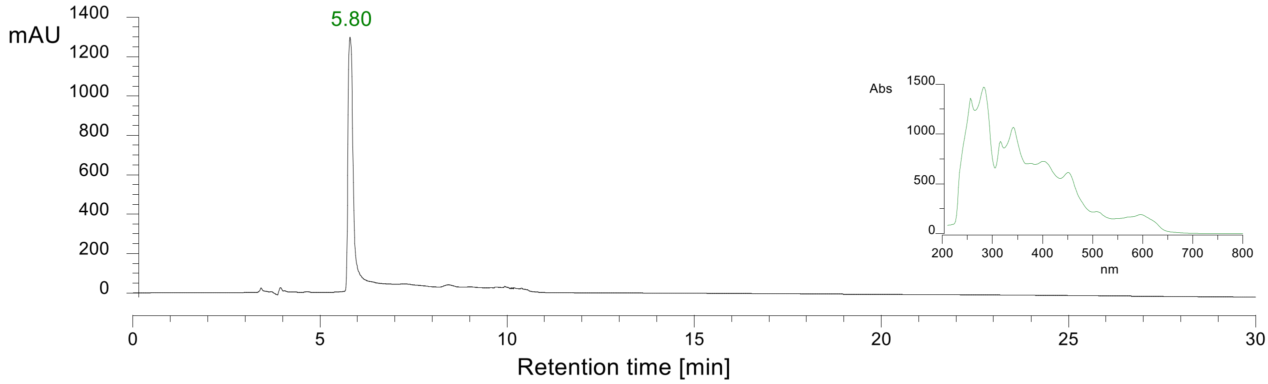

Figure S10. Chiral HPLC analysis of radical **1SO$_2$-a** using CHIRALPAK® IC column monitored at 254 nm.

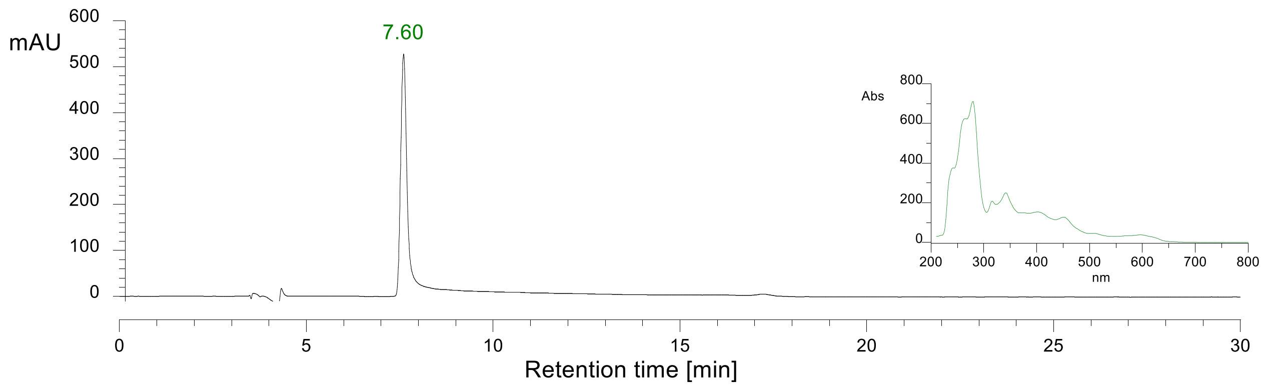

Figure S11. Chiral HPLC analysis of radical **1SO$_2$-b** using CHIRALPAK® IC column monitored at 254 nm.

Preparative separation of enantiomers **1SO-b** was accomplished on a chiral semipreparative column Lux *i*-Cellulose-5 (cellulose tris-3,5-dimethylphenylcarbamate, 250×10 mm) using a CH_2Cl_2/hexane/i-PrOH mixture (50:40:10 ratio) as the liquid phase with a flow rate of 4.0 mL/min and UV detection at 254 nm. Chromatogram is shown in Figure S12.

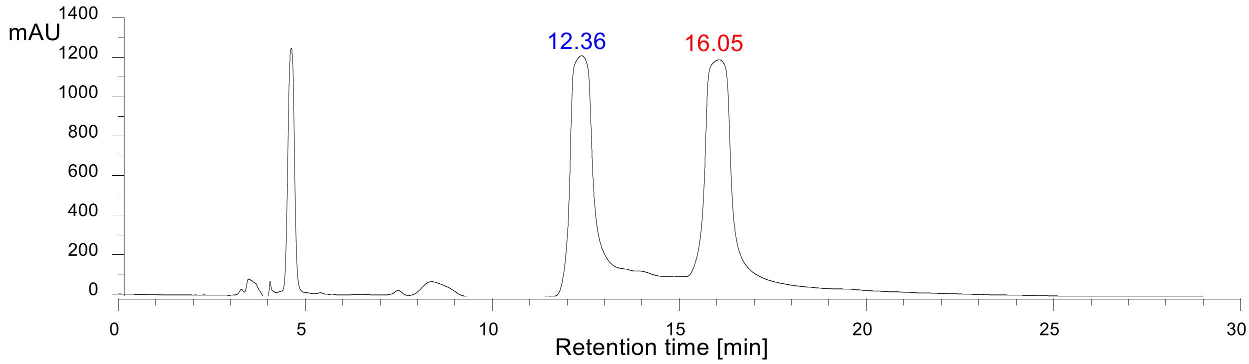

Figure S12. Chiral HPLC analysis of racemic radical **1SO-b** using Lux *i*-Cellulose-5 semipreparative column.

To obtain sufficient quantities of each enantiomer for subsequent studies, several HPLC resolution experiments were conducted. The obtained solutions were stored at ambient temperature for several hours, and then the combined fractions were evaporated to dryness (rotevap, ambient temperature). Analysis of the obtained solid enantiomers by analytical chiral HPLC conducted after several days demonstrated the presence of a single enantiomer without traces of racemization.

5. Electronic circular dichroism spectroscopy

Electronic circular dichroism (ECD) spectra of enantiomers **1SO-b** were recorded on JascoJ-815 CD spectrometer in spectroscopic grade CH_2Cl_2. Results are shown in Figure S13. The notation

fast and *slow* refers to the shorter and longer retention times, respectively, of the individual enantiomers.

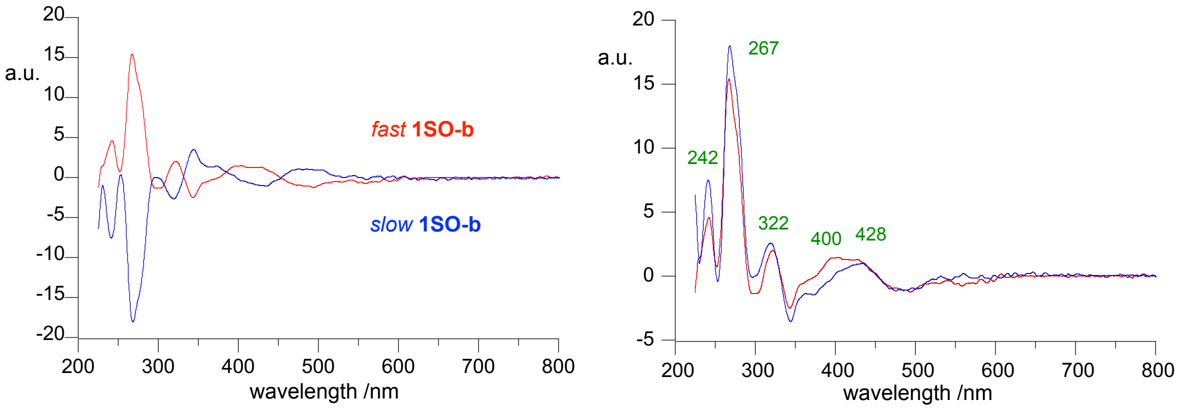

Figure S13. Left: Electronic circular dichroism spectra for the *fast* (red) and the *slow* (blue) enantiomers of radical **1SO-b** in CH_2Cl_2. Right: overlay of the two spectra with listed maxima.

6. Electrochemical results

The electrochemical characterization of selected radicals was conducted using Autolab PGSTAT128N potentiostat/galvanostat instrument in dry and degassed CH_2Cl_2 (concentration 0.5 mM) in the presence of $[n\text{-}Bu_4N]^+[PF_6]^-$ as an electrolyte (concentration 50 mM) using glassy carbon as the working electrode and Ag/AgCl as the reference electrode with a scan rate of 50 mV s^{-1} at *ca.* 20 °C. In the end of each measurement decamethylferrocene ($FcMe_{10}$) was added and the peak potentials were referenced to the $FcMe_{10}/FcMe_{10}^+$ couple. The oxidation potential for the $FcMe_{10}/FcMe_{10}^+$ couple was established at –0.56 V *vs* the Fc/Fc^+ couple (–0.10 V *vs* SCE).[5]

Cyclic voltammetry (CV) plots are shown in Figures S14–S17 and numerical results are shown in Table S3. Correlation of the red-ox potentials with Hammett parameters for model substituents is shown in Figure S18.

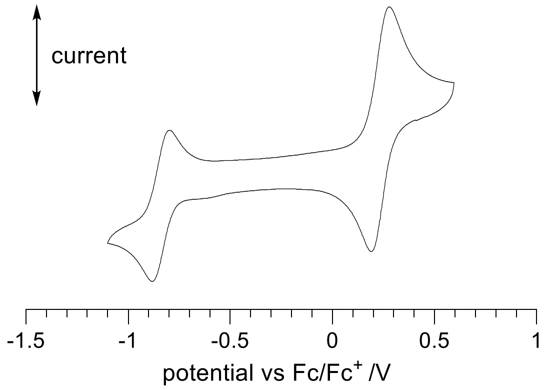

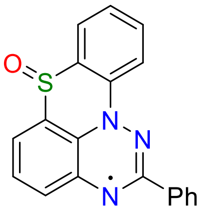

1SO-a

Figure S14. Cyclic voltammogram for **1SO-a**.

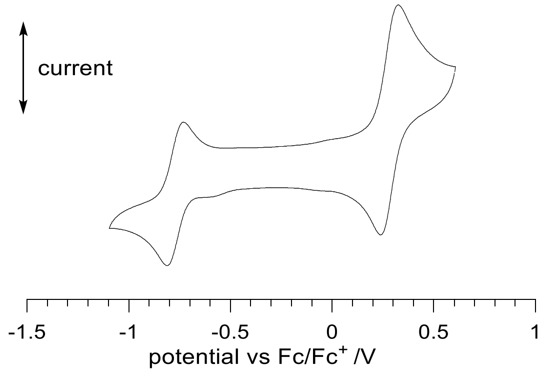

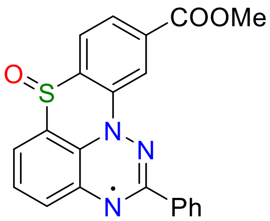

1SO-b

Figure S15. Cyclic voltammogram for **1SO-b**.

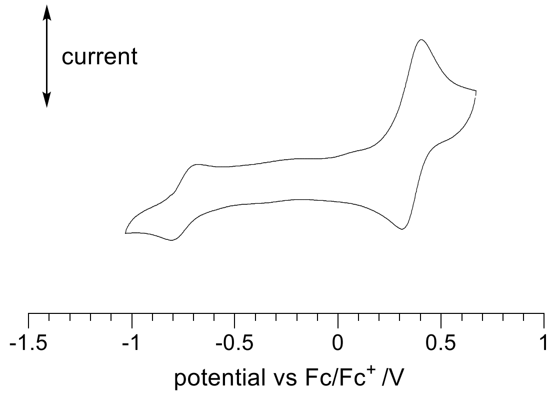

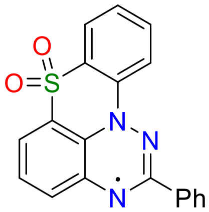

$1SO_2$-a

Figure S16. Cyclic voltammogram for **$1SO_2$-a**.

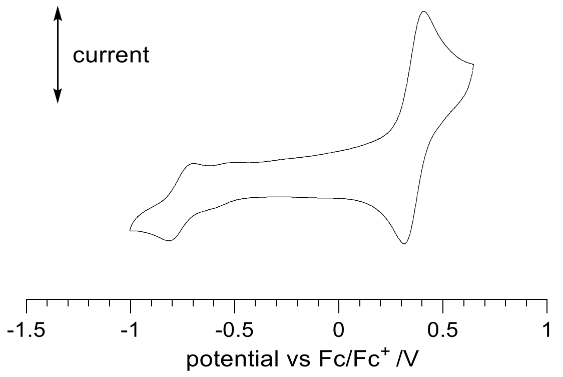

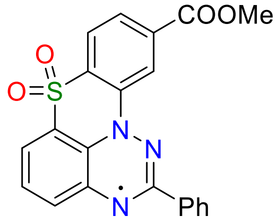

1SO₂-b

Figure S17. Cyclic voltammogram for **1SO₂-b**.

Table S3. Cyclic voltammetry data for selected radicals in CH_2Cl_2.

Radical	$E_{1/2}^{-1/0\ a}$(V)	$E_{1/2}^{0/+1\ a}$ (V)	E_{cell} (V)
**1S-a** [b]	-1.202	-0.112	1.090
**1S-b** [a]	-1.103	-0.047	1.056
**1SO-a**	-0.833	0.232	1.066
**1SO-b**	-0.782	0.280	1.063
**1SO₂-a**	-0.745	0.358	1.103
**1SO₂-b**	-0.756	0.361	1.118

[a] Potential vs Fc/Fc^+ couple. [b]Ref.[1]

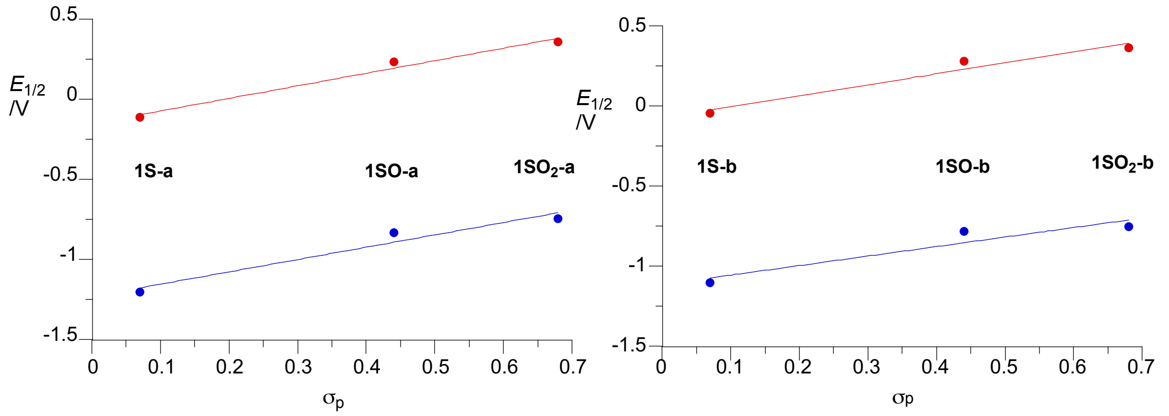

Figure S18. Correlation of red-ox potentials $E_{1/2}$ for radicals **1a** (left) and **1b** (right) with Hammett parameters. Best fitting functions for **1a**: $E_{1/2}^{-1/0}$ = -1.23(8) + 0.77(17)×σ_p, r^2 = 0.95; $E_{1/2}^{0/1}$ = 1.52(5) + 0.78(11)×σ_p, r^2 = 0.98; for **1b**: $E_{1/2}^{-1/0}$ = -1.12(10) + 0.59(21)×σ_p, r^2 = 0.89; $E_{1/2}^{0/1}$ = -0.07(7) + 0.69(15)×σ_p, r^2 = 0.96.

7. EPR spectra

EPR spectra for radicals **1** were recorded on an X-band EMX-Nano EPR spectrometer at ambient temperature on dilute and degassed solutions in distilled benzene in a concentration range of 2-5×10^{-4} M. The microwave power was set with the Power Sweep program below the saturation of the signal, modulation frequency of 100 kHz, modulation amplitude of 0.5 G_{pp} and spectral width of 100 G. Accurate *g*-values were obtained using TEMPO as EMX-Nano internal standard.

Simulations of the spectra were performed with the *EasySpin* (Matlab) using all EPR-active nuclei and DFT results as the starting point for simulations. The chemically equivalent nuclei (H in the Ph substituent) were treated as a group of two identical nuclei. The resulting *hfcc* values were perturbed several times until a global minimum for the fit was achieved. Experimental and simulated spectra are shown in Figures S19–S22 and resulting *hfcc* are listed in Table S4. The *hfcc* values were assigned to the nitrogen nuclei in radicals **1** on the basis of trends in DFT results (*vide infra*).

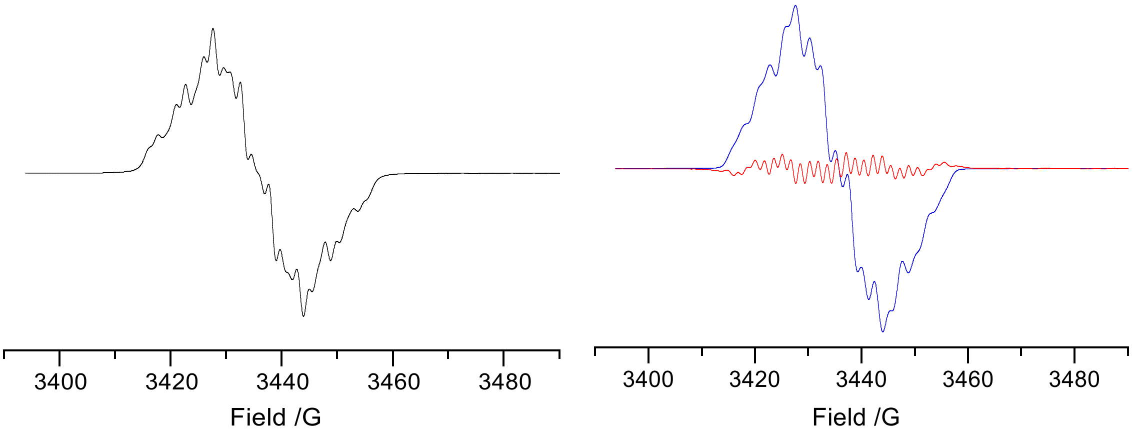

Figure S19. Experimental (black, left), simulated (blue, right) and difference (red, right) spectra for **1SO-a** recorded in CH_2Cl_2 at *ca* 20 °C.

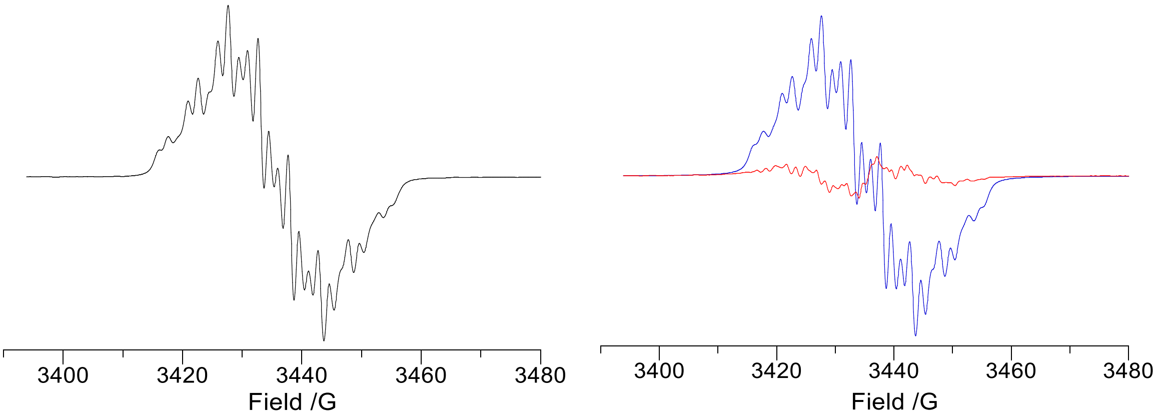

Figure S20. Experimental (black, left), simulated (blue, right) and difference (red, right) spectra for **1SO-b** recorded in benzene at *ca* 20 °C.

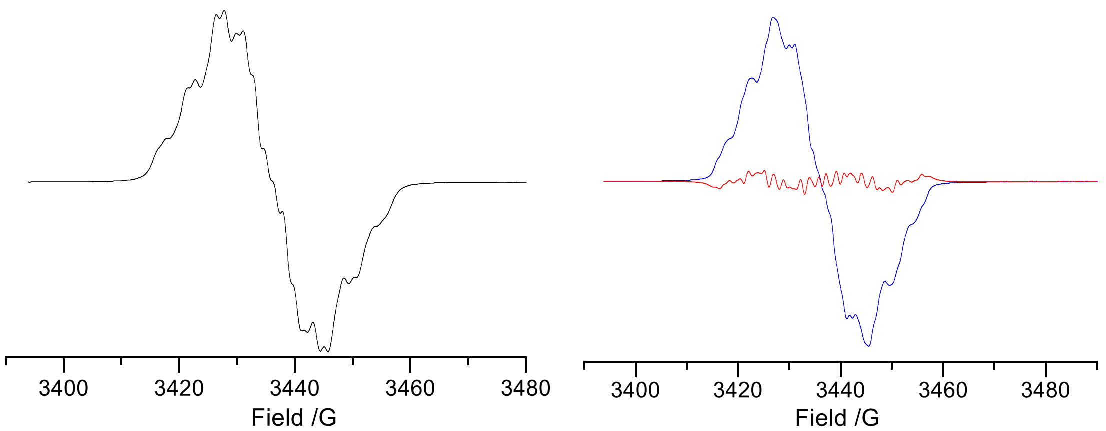

Figure S21. Experimental (black, left), simulated (blue, right) and difference (red, right) spectra for **$1SO_2$-a** recorded in CH_2Cl_2 at *ca* 20 °C.

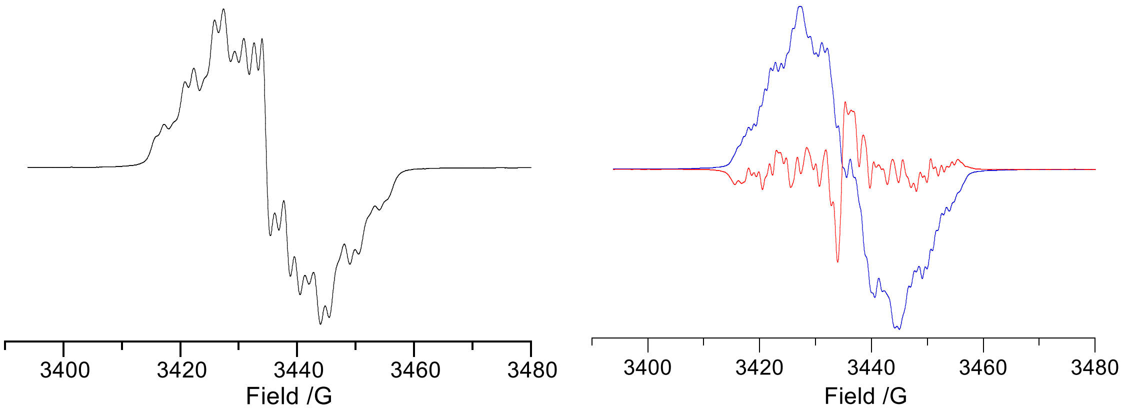

Figure S22. Experimental (black, left), simulated (blue, right) and difference (red, right) spectra for **$1SO_2$-b** recorded in benzene at *ca* 20 °C.

Table S4. Experimental hyperfine coupling constants (G) for radicals in series **1** in benzene at *ca*. 20 °C.[a]

atom	**1S-a** [b]	**1S-b** [b]	**1SO-a**	**1SO-b**	**$1SO_2$-a**	**$1SO_2$-b**
$a_{N(12)}$	7.54	7.08	4.47	4.95	4.49	4.24
$a_{N(1)}$	4.47	4.44	5.25	5.04	5.10	5.06
$a_{N(3)}$	4.49	4.52	4.79	5.00	4.62	4.35
a_H	1.18	1.78	2.96	3.10	3.34	3.89
a_H	0.76	1.47	1.98	2.23	2.34	2.84
a_H	0.74	1.36	1.74	1.55	1.83	1.78
a_H	0.72	0.86	1.73	1.43	1.31	1.78
a_H	0.71	0.74	0.85	0.53	0.70	1.07
a_H	0.69	0.28	0.80	0.58	0.68	0.95
a_H	0.53	0.35	0.72	0.47	0.64	0.93
a_H	0.40	-	0.67	-	0.62	-
2x a_H	0.31	0.11	0.42	0.16	0.38	0.68
2x a_H	0.26	0.06	0.31	0.35	0.24	0.79
g	2.0039	2.0043	2.0042	2.0043	2.0040	2.0045

[a] Assignment based on DFT *hfcc*. [b] From reference.[1]

A correlation of the experimental *hfcc* with Hammett parameters[6] for model substituents, PhS (σ_p = 0.07), PhSO (σ_p = 0.44), and $PhSO_2$ (σ_p = 0.68) is shown in Figure S23.

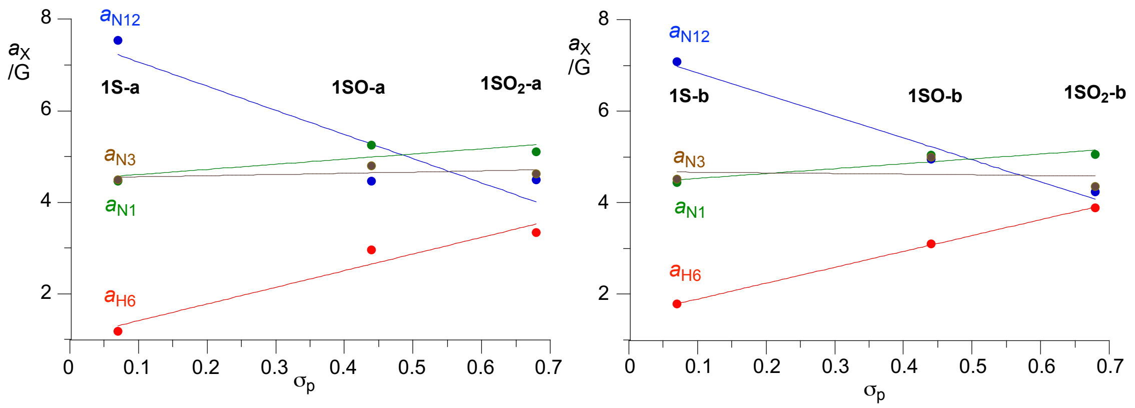

Figure S23. Correlation of experimental *hfcc* a_X for radicals **1a** (left) and **1b** (right) with Hammett parameters. Best fitting functions for **1a**: $a_{N12} = 7.6(11) - 5.3(22)\times\sigma_p$, $r^2 = 0.84$; $a_{H6} = 1.0(4) + 3.6(9)\times\sigma_p$, $r^2 = 0.95$; for **1b**: $a_{N12} = 7.3(3) - 4.7(7)\times\sigma_p$, $r^2 = 0.98$; $a_{H6} = 1.55(4) + 3.47(7)\times\sigma_p$, $r^2 = 0.999$.

8. Computational details and results

Quantum-mechanical calculations were carried out using Gaussian 16 suite of programs.[7] Geometry optimizations were undertaken at the B3LYP/6-311G(2d,p) level of theory using tight convergence limits and without symmetry constraints (C1).

Full computational data for this project are available at Zenodo repository (DOI: zenodo.org/10.5281/zenodo.19729818).

a) isotropic Fermi contact coupling constants (hfcc)

Isotropic Fermi contact coupling constants for radicals **1** and the Blatter radical were calculated using the CAM-B3LYP/gen // UB3LYP/6-311G(2d,p) method in benzene dielectric medium requested with the SCRF(Solvent=Benzene) keyword (PCM model).[8] These calculations used the EPRIII basis set for C, H, N, O atoms and 6-311+G(2df) basis set for S atom requested with the “Gen” keyword.

The resulting *hfcc* values are shown in Table S5 and spin densities are listed in Table S6. A comparison of the experimental and DFT-derived major *hfcc* is shown in Figure S24.

Spin density maps of radicals **1SO** and **$1SO_2$** are shown in Figure S25.

Table S5. Calculated hyperfine coupling constants (G) of radicals **1** in benzene at the UCAM-B3LYP/Gen // UB3LYP/6-311G(2d,p) level of theory.[a]

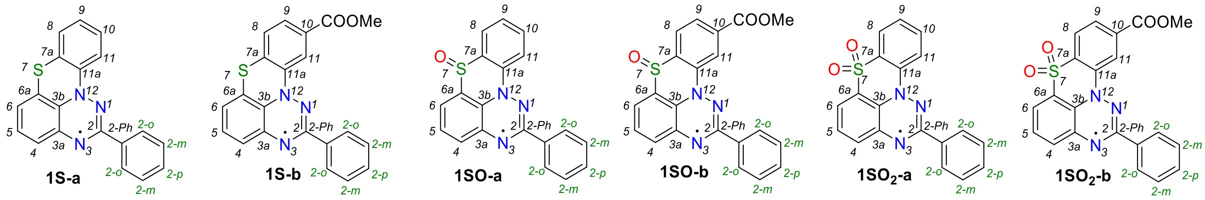

hfcc/G	1S-a	1S-b	1SO-a	1SO-b	$1SO_2$-a	$1SO_2$-b
$a_{N(12)}$	6.10	5.98	4.41	4.29	4.45	4.33
$a_{N(1)}$	4.19	4.25	4.70	4.76	4.80	4.86
$a_{N(3)}$	4.33	4.36	4.64	4.67	4.68	4.71
$a_{H(4)}$	-1.15	-1.20	-2.84	-2.89	-2.92	-2.96
$a_{H(5)}$	-0.43	-0.38	0.23	0.29	0.38	0.44
$a_{H(6)}$	-1.59	-1.67	-3.59	-3.67	-3.77	-3.84
$a_{H(8)}$	1.02	0.98	0.89	0.91	0.86	0.89
$a_{H(9)}$	-2.04	-1.92	-1.79	-1.87	-1.73	-1.80
$a_{H(10)}$	1.17	–	0.99	–	0.98	–
$a_{H(11)}$	-2.13	-2.25	-1.85	-1.86	-1.81	–
$a_{H(3\text{-}o)\ avg}$	0.58	0.60	0.70	0.71	0.72	0.73
$a_{H(3\text{-}m)\ avg}$	-0.32	-0.33	-0.38	-0.39	-0.39	-0.39
$a_{H(3\text{-}p)}$	0.49	0.50	0.60	0.61	0.61	0.62

[a] EPRIII basis set for C, H, N, O atoms and 6-311+G(2df) for S atom.

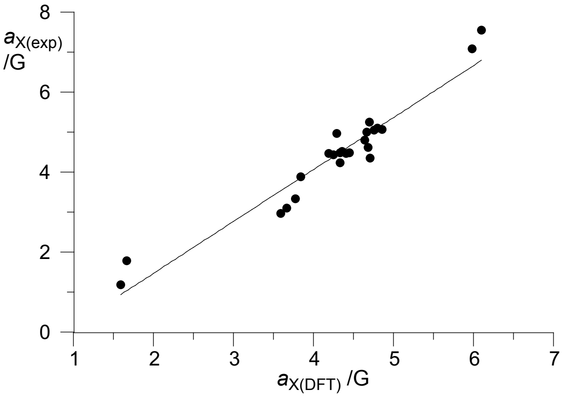

Figure S24. Correlation of experimental and DFT-derived major *hfcc* for N(12), N(1), N(3) and H(6) for radicals **1a** and **1b**. Best fitting line: $a_{x(exp)} = -1.1(3)+1.30(8)\times a_{x(DFT)}$, $r^2 = 0.92$.

Table S6. Calculated spin densities of radicals **1** in benzene at the UCAM-B3LYP/Gen // UB3LYP/6-311G(2d,p) level of theory.[a]

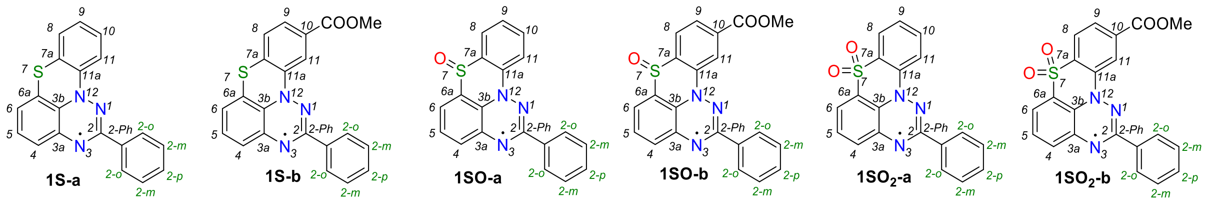

Spin density	**1S-a**	**1S-b**	**1SO-a**	**1SO-b**	**1SO$_2$-a**	**1SO$_2$-b**
$\rho_{N(12)}$	0.235	0.229	0.182	0.176	0.183	0.176
$\rho_{N(1)}$	0.301	0.308	0.325	0.330	0.330	0.336
$\rho_{N(3)}$	0.295	0.298	0.319	0.320	0.322	0.324
$\rho_{C(2)}$	-0.075	-0.080	-0.088	-0.091	-0.092	-0.094
$\rho_{C(3a)}$	0.002	0.001	0.096	-0.014	-0.017	-0.018
$\rho_{C(3b)}$	0.078	0.083	0.048	0.050	0.052	0.053
$\rho_{C(4)}$	0.030	0.032	0.096	0.099	0.099	0.101
$\rho_{C(5)}$	0.008	0.008	-0.018	-0.019	-0.026	-0.027
$\rho_{C(6)}$	0.055	0.057	0.133	0.134	0.141	0.142
$\rho_{C(6a)}$	0.003	0.001	-0.027	-0.030	-0.036	-0.038
$\rho_{S(7)}$	0.030	0.031	-0.002	-0.001	0.001	0.001
$\rho_{C(7a)}$	0.077	0.071	0.067	0.069	0.067	0.068
$\rho_{C(8)}$	-0.047	-0.044	-0.038	-0.039	-0.037	-0.038
$\rho_{C(9)}$	0.077	0.069	0.067	0.069	0.064	0.067
$\rho_{C(10)}$	-0.049	-0.040	-0.040	-0.037	-0.038	-0.035
$\rho_{C(11)}$	0.072	0.066	0.067	0.057	0.064	0.054
$\rho_{C(11a)}$	-0.061	-0.054	-0.048	-0.040	-0.045	-0.037
$\rho_{C(2\text{-}Ph)}$	0.010	0.010	0.013	0.013	0.014	0.013
$\rho_{C(3\text{-}o)}$ avg	-0.023	-0.023	-0.027	-0.027	-0.027	-0.028
$\rho_{C(3\text{-}m)}$ avg	0.012	0.012	0.015	0.015	0.015	0.016
$\rho_{C(3\text{-}p)}$	-0.019	0.020	-0.023	-0.024	-0.024	-0.024

[a] EPRIII basis set for C, H, N, O atoms and 6-311+G(2df) for S atom.

b) spin delocalization of radicals in benzene dielectric medium

Spin delocalization parameter RDV (Radical Delocalization Value)[9] was calculated according to the formula:

$$RDV = \sum_{i=1}^{n} (\rho_i)^2$$

where spin concentration ρ_i on heavy atoms *i* (hydrogen atoms summed up to heavy atoms) is obtained with the UCAM-B3LYP/Gen // UB3LYP/6-311G(2d,p) method in benzene dielectric mediums using the PCM model.[8] These calculations used the EPRIII basis set for C, H, N, O atoms and 6-311+G(2df)

basis set for S atom requested with the "Gen" keyword. For the purpose of this work, an inverse is reported: $RDV^{-1}=1/RDV$, since now larger values corresponds to greater delocalization. Results are shown in Table S7.

Table S7. Radical delocalization value (RDV^{-1}) for radicals **1**.

Radical	RDV^{-1} (in benzene)
**1S-a**	3.618
**1S-b**	3.615
**1SO-a**	3.340
**1SO-b**	3.305
**1SO2-a**	3.232
**1SO2-b**	3.205

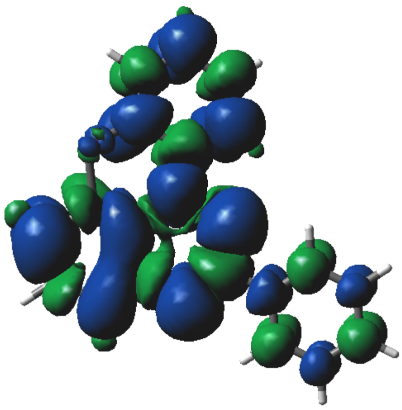

1SO-a

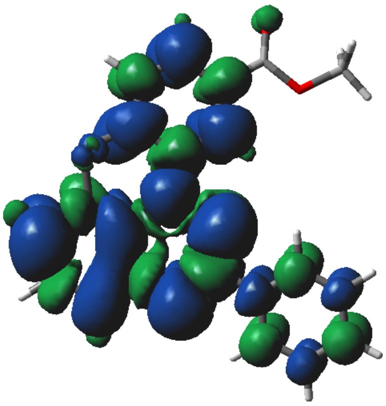

1SO-b

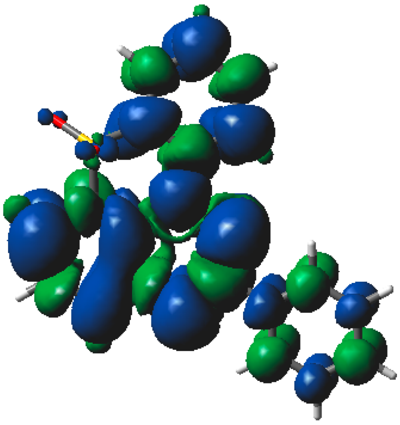

$1SO_2$-a

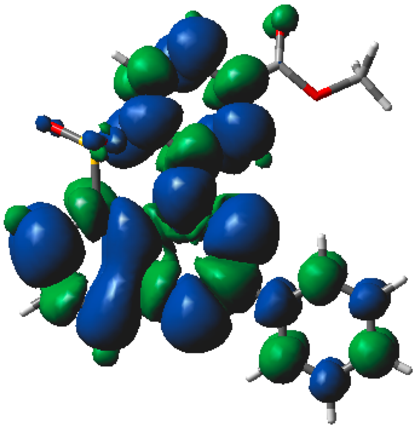

$1SO_2$-b

Figure S25. Spin density maps of radicals **1SO** and **$1SO_2$** at the UCAM-B3LYP/Gen // UB3LYP/6-311G(2d,p) level of theory in benzene dielectric medium. EPRIII basis set for C, H, N, O atoms and 6-311+G(2df) for S atom. *Isovalue*: MO = 0.020, Density = 0.0004.

c) magnetic exchange interactions in the crystal structure

The spin-spin exchange interaction J_{DFT} for close pairs of molecules in the crystal lattice of **1SO-b** was determined using the Yamaguchi formalism:[10]

$$J_{DFT} = \frac{E_{BS} - E_T}{\langle S^2 \rangle_T - \langle S^2 \rangle_{BS}}$$

where the SCF energies of the triplet (E_T) and broken symmetry singlet (E_{BS}) and total spin angular momenta $\langle S^2 \rangle$ before spin annihilation were obtained by single point calculations for selected pairs of molecules at crystallographically determined coordinates at the UB3LYP/6-311+G(2d,p) level of theory. The input geometries and calculated exchange interaction energies are shown in Figure S26.

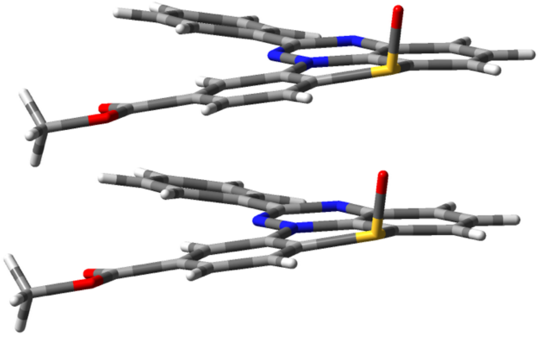

crystallographic close pair J_{DFT} = -55.3 cal mol^{-1}

Figure S26. Pairs of molecules of radical **1SO-b** in their crystallographic coordinates used for DFT calculations and the resulting unprojected spin-spin interaction energies.

d) electronic excitations

Electronic excitation energies in CH_2Cl_2 dielectric medium were obtained at the UB3LYP/6-311+G(2d,p) // UB3LYP/6-311G(2d,p) level of theory using the time-dependent TD-DFT method[11] supplied in the Gaussian 16 package. Solvation models in calculations were implemented with the PCM model[8] using the SCRF(solvent=CH_2Cl_2) keyword. Four lowest excitation energies, classified as $\pi \rightarrow \pi^*$ transitions for radicals **1** are listed in Table S8.

Energies of FMOs involved in the low energy transitions in radicals **1** are listed in Table S9. Graphical representations of the relevant MO are shown in Figures S27–S30.

Table S8. Major low energy electronic excitation and oscillator strength values with the indicated main electronic transitions for radicals **1** obtained at the TD-UB3LYP/6-311G+(2d,p) // UB3LYP/6-311G(2d,p) level of theory in CH_2Cl_2 dielectric medium.

Radical	$D_0 \rightarrow D_1$, (π→π*) λ$_{max}$ /nm (*f*) α-HOMO→α-LUMO and β-HOMO→β-LUMO	$D_0 \rightarrow D_2$, (π→π*) λ$_{max}$ /nm (*f*) α-HOMO→α-LUMO and β-HOMO→β-LUMO	$D_0 \rightarrow D_3$, π→π* λ$_{max}$ /nm (*f*) α-HOMO→α-LUMO+1
**1S-a**	650.4 (0.045), 0% and 96%	591.2 (0.004), 92% and 0%	466.2 (0.029), 90%
**1S-b**	642.5 (0.040), 3% and 94%	605.8 (0.0004), 72% and 1%	532.0 (0.019), 74%
**1SO-a**	563.1 (0.008), 61% and 34%	510.4 (0.032), 29% and 54%	443.0(0.047),[a] 88%
**1SO-b**	565.9 (0.011), 38% and 33%	526.1 (0.013), 65% and 28%	504.8 (0.031), 51%
**1SO$_2$-a**	562.7 (0.013), 47% and 48%	510.2 (0.025), 42% and 41%	434.9 (0.060),[a] 85%
**1SO$_2$-b**	566.3 (0.016), 19% and 46%	524.3 (0.016), 58% and 32%	493.4 (0.029) 56%,[b]

[a] $D_0 \rightarrow D_4$ state. [b] 19% α-HOMO→α-LUMO.

Table S9. Energies of MO involved in low energy transitions in radicals **1** obtained using the UB3LYP/6-311+G(2d,p)//UB3LYP/6-311G(2d,p) method in CH_2Cl_2 dielectric medium.

Radical	α-HOMO π /eV	α-LUMO π* /eV	α-LUMO+1 π* /eV	β-HOMO-1 π /eV	β-HOMO π* /eV	β-LUMO π* /eV
**1S-a**	-4.967	-1.816	-1.326	-6.852	-5.893	-3.121
**1S-b**	-5.053	-2.070	-1.799	-6.903	-6.005	-3.196
**1SO-a**	-5.336	-1.982	-1.652	-7.071	-6.725	-3.510
**1SO-b**	-5.411	-2.329	-1.997	-7.158	-6.775	-3.586
**1SO$_2$-a**	-5.465	-2.068	-1.700	-7.300	-6.789	-3.615
**1SO$_2$-b**	-5.538	-2.409	-2.061	-7.321	-6.833	-3.689

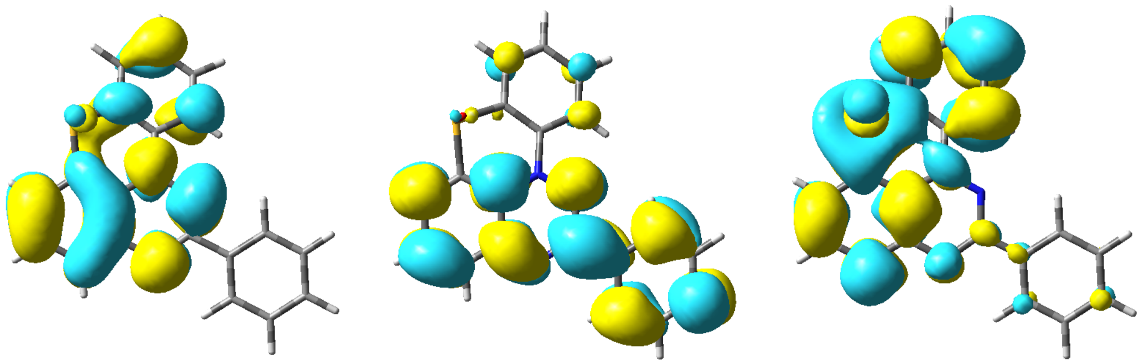

$E_{\alpha\text{-HOMO}}$ = -5.336 eV $E_{\alpha\text{-LUMO}}$ = -1.982 eV $E_{\alpha\text{-LUMO+1}}$ = -1.652 eV

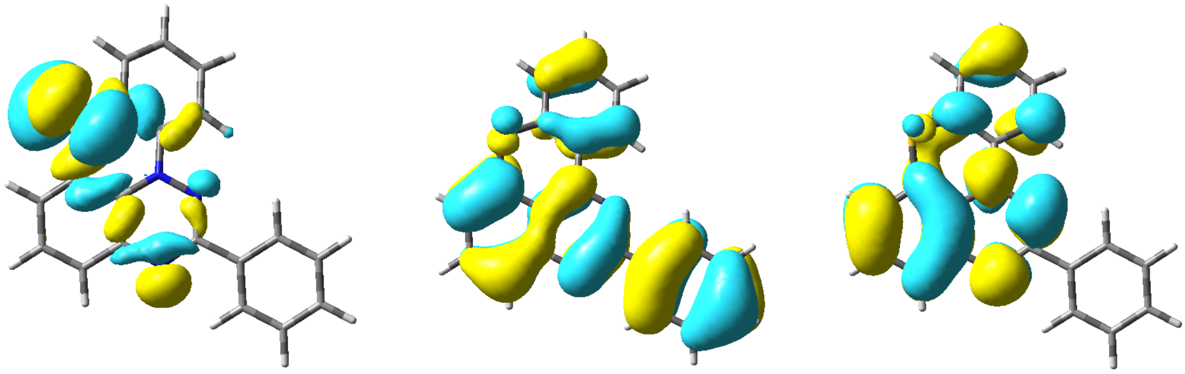

$E_{\beta\text{-HOMO-1}}$ = -7.071 eV $E_{\beta\text{-HOMO}}$ = -6.725 eV $E_{\beta\text{-LUMO}}$ = -3.510 eV

Figure S27. UB3LYP/6-311+G(2d,p)//UB3LYP/6-311G(2d,p) derived contours and energies of molecular orbitals relevant to low energy excitations of **1SO-a** in CH_2Cl_2 dielectric medium.

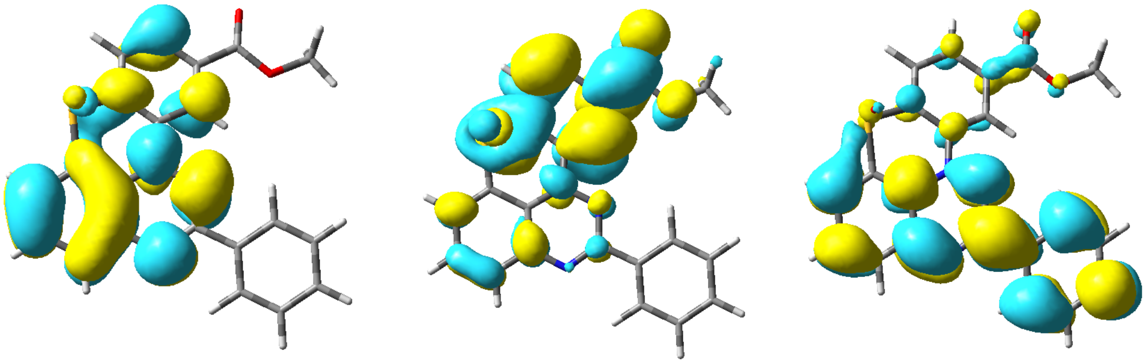

$E_{\alpha\text{-HOMO}}$ = -5.411 eV $E_{\alpha\text{-LUMO}}$ = -2.329 eV $E_{\alpha\text{-LUMO+1}}$ = -1.997 eV

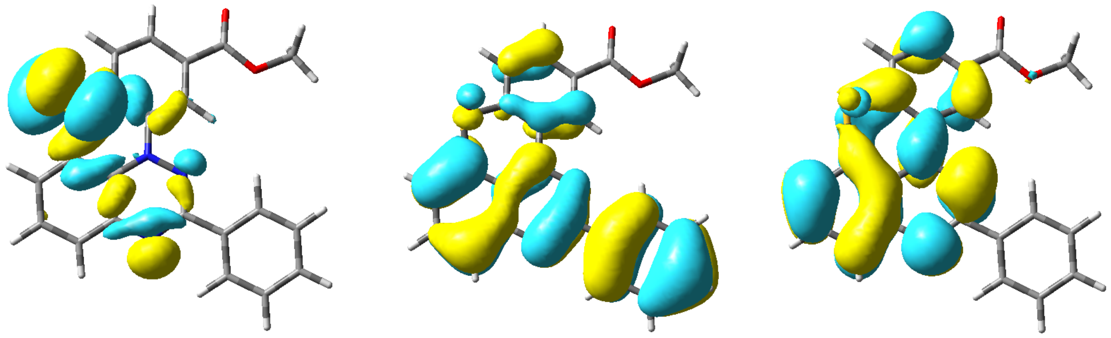

$E_{\beta\text{-HOMO-1}}$ = -7.158 eV $E_{\beta\text{-HOMO}}$ = -6.775 eV $E_{\beta\text{-LUMO}}$ = -3.586 eV

Figure S28. UB3LYP/6-311+G(2d,p)//UB3LYP/6-311G(2d,p) derived contours and energies of molecular orbitals relevant to low energy excitations of **1SO-b** in CH_2Cl_2 dielectric medium.

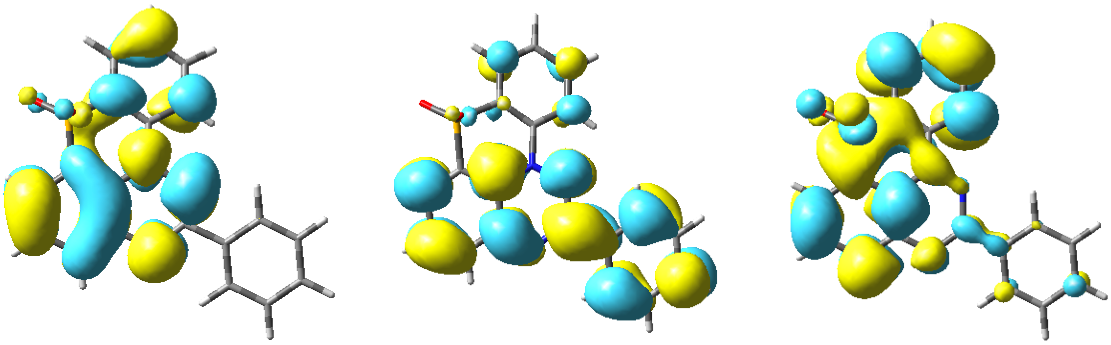

$E_{\alpha\text{-HOMO}}$ = -5.465 eV $E_{\alpha\text{-LUMO}}$ = -2.068 eV $E_{\alpha\text{-LUMO+1}}$ = -1.700 eV

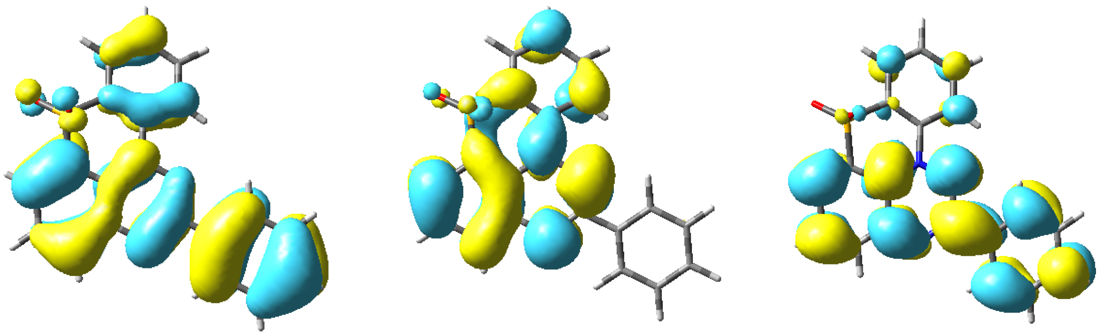

$E_{\beta\text{-HOMO-1}}$ = -7.300 eV $E_{\beta\text{-HOMO}}$ = -6.789 eV $E_{\beta\text{-LUMO}}$ = -3.615 eV

Figure S29. UB3LYP/6-311+G(2d,p)//UB3LYP/6-311G(2d,p) derived contours and energies of molecular orbitals relevant to low energy excitations of **$1SO_2$-a** in CH_2Cl_2 dielectric medium.

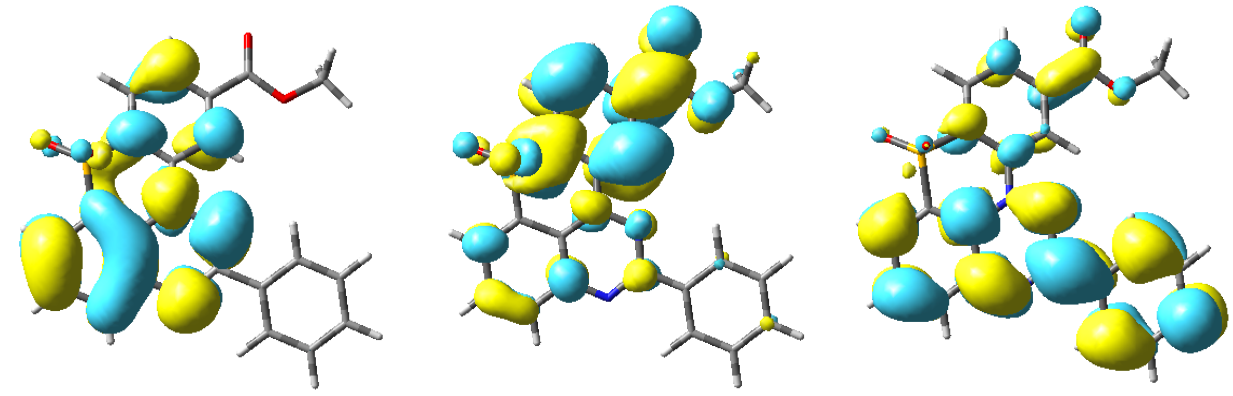

$E_{\alpha\text{-HOMO}}$ = -5.538 eV $E_{\alpha\text{-LUMO}}$ = -2.409 eV $E_{\alpha\text{-LUMO+1}}$ = -2.061 eV

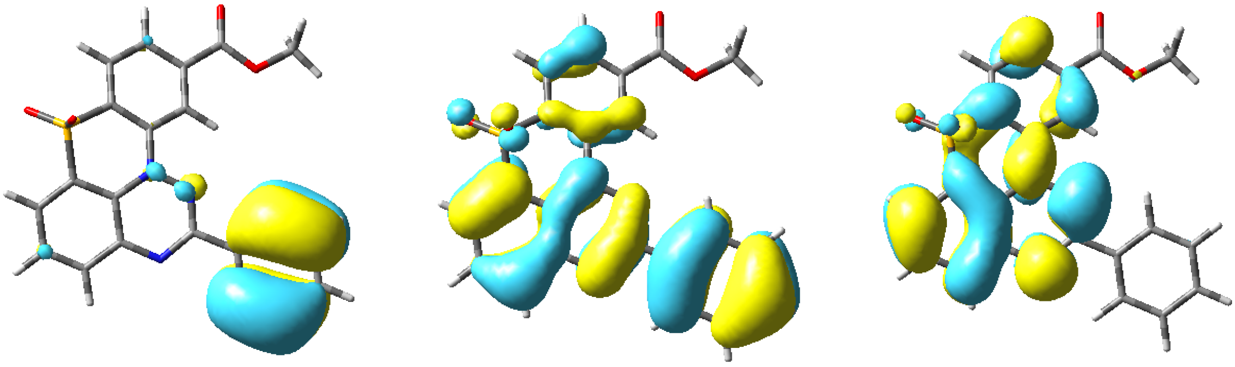

$E_{\beta\text{-HOMO-1}}$ = -7.321eV $E_{\beta\text{-HOMO}}$ = -6.833 eV $E_{\beta\text{-LUMO}}$ = -3.689 eV

Figure S30. UB3LYP/6-311+G(2d,p)//UB3LYP/6-311G(2d,p) derived contours and energies of molecular orbitals relevant to low energy excitations of **$1SO_2$-b** in CH_2Cl_2 dielectric medium.

e) partial output data from TD-DFT calculations for radicals

Method: UB3LYP/6-311+G(2d,p)// UB3LYP/6-311G(2d,p)

Keywords: TD(nstates=20, root=1) SCRF(solvent=CH2Cl2) SCF=tight

1S-a

```
Excited State   1:  2.086-A      1.9063 eV  650.40 nm  f=0.0445  <S**2>=0.838
     81B -> 82B        0.97895
 This state for optimization and/or second-order correction.
 Total Energy, E(TD-HF/TD-DFT) =  -1293.87225092
 Copying the excited state density for this state as the 1-particle RhoCI
density.

 Excited State   2:  2.041-A      2.0973 eV  591.15 nm  f=0.0040  <S**2>=0.792
     82A -> 83A        0.95812
     82A -> 84A        0.10168
     80B -> 82B       -0.14661

 Excited State   3:  2.190-A      2.6595 eV  466.19 nm  f=0.0289  <S**2>=0.949
     81A -> 84A        0.12792
     82A -> 84A        0.94940

 Excited State   4:  2.044-A      2.8230 eV  439.20 nm  f=0.0070  <S**2>=0.795
     76B -> 82B        0.10067
     77B -> 82B        0.87039
     78B -> 82B        0.11355
     80B -> 82B        0.41620

 Excited State   5:  2.110-A      2.9694 eV  417.54 nm  f=0.0352  <S**2>=0.863
     81A -> 83A        0.12549
     82A -> 83A        0.14249
     77B -> 82B       -0.43358
     80B -> 82B        0.84399
```

1S-b

```
Excited State   1:  2.090-A      1.9298 eV  642.47 nm  f=0.0403  <S**2>=0.842
     97A -> 98A        0.16263
     96B -> 97B        0.96507
 This state for optimization and/or second-order correction.
 Total Energy, E(TD-HF/TD-DFT) =  -1521.82614835
 Copying the excited state density for this state as the 1-particle RhoCI
density.

 Excited State   2:  2.086-A      2.0466 eV  605.79 nm  f=0.0004  <S**2>=0.838
     96A -> 99A       -0.10087
     97A -> 98A        0.85621
     97A -> 99A       -0.42408
     97A ->100A       -0.12184
     95B -> 97B        0.13380
     96B -> 97B       -0.10356

 Excited State   3:  2.140-A      2.3307 eV  531.95 nm  f=0.0185  <S**2>=0.895
     96A -> 98A       -0.12961
     97A -> 98A        0.41279
     97A -> 99A        0.85683
     96B -> 97B       -0.14658
     96B -> 98B        0.10454
```

```
 Excited State   4:  2.050-A      2.8002 eV  442.77 nm  f=0.0012  <S**2>=0.800
     97A ->100A        0.16401
     92B -> 97B       -0.26827
     93B -> 97B        0.85170
     95B -> 97B        0.37193

 Excited State   5:  2.147-A      2.8686 eV  432.22 nm  f=0.0358  <S**2>=0.902
     97A -> 98A        0.13638
     97A ->100A        0.92608
     92B -> 97B        0.21252
     93B -> 97B       -0.12403
```

1SO-a

```
Excited State   1:  2.056-A      2.2019 eV  563.07 nm  f=0.0083  <S**2>=0.807
     86A -> 87A        0.78257
     85B -> 86B       -0.58384
 This state for optimization and/or second-order correction.
 Total Energy, E(TD-HF/TD-DFT) =  -1369.07255824
 Copying the excited state density for this state as the 1-particle RhoCI
density.

 Excited State   2:  2.057-A      2.4294 eV  510.35 nm  f=0.0321  <S**2>=0.807
     86A -> 87A        0.54216
     86A -> 88A       -0.22167
     86A -> 89A        0.16233
     86A -> 92A        0.10162
     82B -> 86B        0.19691
     83B -> 86B        0.11584
     85B -> 86B        0.73516

 Excited State   3:  2.047-A      2.5631 eV  483.73 nm  f=0.0008  <S**2>=0.797
     79B -> 86B       -0.12020
     80B -> 86B        0.41691
     81B -> 86B        0.51769
     82B -> 86B        0.12014
     84B -> 86B        0.69275

 Excited State   4:  2.077-A      2.7988 eV  443.00 nm  f=0.0474  <S**2>=0.829
     86A -> 87A        0.14292
     86A -> 88A        0.93851
     81B -> 86B        0.11096
     85B -> 86B        0.16634

 Excited State   5:  2.078-A      2.8680 eV  432.30 nm  f=0.0095  <S**2>=0.829
     80B -> 86B       -0.39184
     81B -> 86B       -0.44397
     82B -> 86B       -0.27815
     83B -> 86B       -0.20912
     84B -> 86B        0.69161
```

1SO-b

```
Excited State   1:  2.057-A      2.1908 eV  565.94 nm  f=0.0128  <S**2>=0.808
    101A ->102A       -0.49069
```

```
    101A ->103A        0.61563
    100B ->101B       -0.57665
 This state for optimization and/or second-order correction.
 Total Energy, E(TD-HF/TD-DFT) =  -1597.02549388
 Copying the excited state density for this state as the 1-particle RhoCI
density.

 Excited State   2:  2.099-A      2.3568 eV  526.08 nm  f=0.0129  <S**2>=0.852
    101A ->102A        0.80573
    101A ->103A        0.14526
     97B ->101B       -0.11289
    100B ->101B       -0.52748

 Excited State   3:  2.050-A      2.4561 eV  504.80 nm  f=0.0313  <S**2>=0.801
    101A ->102A        0.26225
    101A ->103A        0.71434
    101A ->104A        0.26091
    101A ->107A        0.10115
     97B ->101B        0.20481
    100B ->101B        0.51615

 Excited State   4:  2.049-A      2.5486 eV  486.48 nm  f=0.0010  <S**2>=0.799
     94B ->101B       -0.10183
     95B ->101B       -0.12831
     96B ->101B        0.65007
     97B ->101B        0.16930
     99B ->101B        0.68179

 Excited State   5:  2.100-A      2.8652 eV  432.73 nm  f=0.0114  <S**2>=0.852
     95B ->101B        0.13046
     96B ->101B       -0.52736
     97B ->101B       -0.37028
     98B ->101B        0.17014
     99B ->101B        0.68903
    100B ->101B        0.11612

 Excited State   6:  2.153-A      2.9291 eV  423.29 nm  f=0.0889  <S**2>=0.909
    101A ->103A       -0.16639
    101A ->104A        0.88454
     95B ->101B        0.15755
     96B ->101B       -0.11263
     97B ->101B        0.11877
    100B ->101B       -0.22268
```

$1SO_2$-a

```
Excited State   1:  2.062-A      2.2035 eV  562.66 nm  f=0.0125  <S**2>=0.813
     90A -> 91A        0.68674
     89B -> 90B        0.69214
 This state for optimization and/or second-order correction.
 Total Energy, E(TD-HF/TD-DFT) =  -1444.31559528
 Copying the excited state density for this state as the 1-particle RhoCI
density.

 Excited State   2:  2.053-A      2.4300 eV  510.23 nm  f=0.0251  <S**2>=0.803
     90A -> 91A        0.65039
     90A -> 92A       -0.22987
     90A -> 93A        0.12807
     90A -> 96A       -0.10171
```

```
     87B -> 90B        0.23251
     89B -> 90B       -0.63965

 Excited State   3:  2.042-A      2.6058 eV  475.81 nm  f=0.0014  <S**2>=0.792
     84B -> 90B       -0.14840
     86B -> 90B        0.92391
     87B -> 90B       -0.25512
     89B -> 90B       -0.10604

 Excited State   4:  2.086-A      2.8507 eV  434.92 nm  f=0.0604  <S**2>=0.838
     90A -> 91A        0.18639
     90A -> 92A        0.92517
     83B -> 90B        0.13495
     89B -> 90B       -0.17317

 Excited State   5:  2.107-A      2.9695 eV  417.53 nm  f=0.0658  <S**2>=0.860
     90A -> 91A       -0.14083
     90A -> 92A        0.14204
     90A -> 94A       -0.10497
     84B -> 90B       -0.11431
     86B -> 90B        0.25239
     87B -> 90B        0.72027
     88B -> 90B       -0.51346
     89B -> 90B        0.16726

 Excited State   6:  2.084-A      3.1040 eV  399.43 nm  f=0.0027  <S**2>=0.836
     87B -> 90B        0.51882
     88B -> 90B        0.82711
```

1SO$_2$-b

```
Excited State   1:  2.061-A      2.1894 eV  566.30 nm  f=0.0159  <S**2>=0.811
    105A ->106A        0.43361
    105A ->107A       -0.53994
    104B ->105B        0.68746
 This state for optimization and/or second-order correction.
 Total Energy, E(TD-HF/TD-DFT) =  -1672.26816627
 Copying the excited state density for this state as the 1-particle RhoCI
density.

 Excited State   2:  2.091-A      2.3650 eV  524.26 nm  f=0.0109  <S**2>=0.843
    105A ->106A        0.76573
    105A ->107A       -0.11979
    105A ->108A        0.12188
    102B ->105B        0.17386
    104B ->105B       -0.56491

 Excited State   3:  2.062-A      2.5127 eV  493.44 nm  f=0.0287  <S**2>=0.813
    105A ->106A        0.42644
    105A ->107A        0.75224
    105A ->108A       -0.25890
    101B ->105B       -0.13275
    102B ->105B       -0.20930
    104B ->105B        0.29249
```

```
Excited State   4:  2.043-A      2.5887 eV  478.95 nm  f=0.0016  <S**2>=0.793
   105A ->107A        0.14673
   101B ->105B        0.90027
   102B ->105B        0.29770
   104B ->105B        0.13039

Excited State   5:  2.143-A      2.9021 eV  427.22 nm  f=0.0815  <S**2>=0.899
   100A ->106A       -0.10250
   105A ->107A        0.23699
   105A ->108A        0.82803
    97B ->105B       -0.10232
   101B ->105B       -0.14052
   102B ->105B        0.29766
   104B ->105B        0.25135

Excited State   6:  2.123-A      2.9670 eV  417.88 nm  f=0.0821  <S**2>=0.876
   105A ->108A       -0.35141
   105A ->109A       -0.11001
    97B ->105B        0.10256
    99B ->105B       -0.11688
   101B ->105B       -0.25968
   102B ->105B        0.59944
   103B ->105B       -0.57745
   104B ->107B       -0.1059
```

f) determination of absolute configuration of enantiomers

The absolute configuration was assigned to radical enantiomers **1SO-b** by comparison of experimental and DFT calculated electronic circular dichroism (ECD) spectra. Theoretical ECD spectra were obtained at the UB3LYP/6-311+G(2d,p) // UB3LYP/6-311G(2d,p) level of theory in CH_2Cl_2 dielectric medium using the PCM model requested with SCRF(solvent=CH2Cl2) keyword, with TD method and 90 states. Results are shown in Figures S31 and S32. UV-vis peaks half-width at half-height set at 0.1 eV $\Delta\varepsilon$, rotatory strengths (R_{vel} /10^{-40} esu^{2}cm^2).

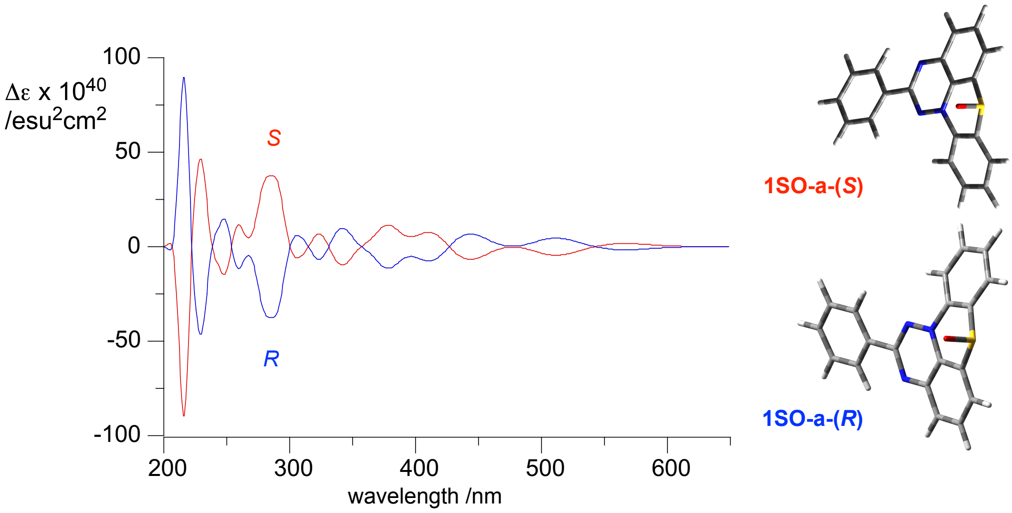

Figure S31. Left: Calculated (DFT) electronic circular dichroism spectra for two enantiomers of **1SO-a** in CH_2Cl_2 dielectric medium. Right: UB3LYP/6-311G(2d,p) optimized structures of the two enantiomers.

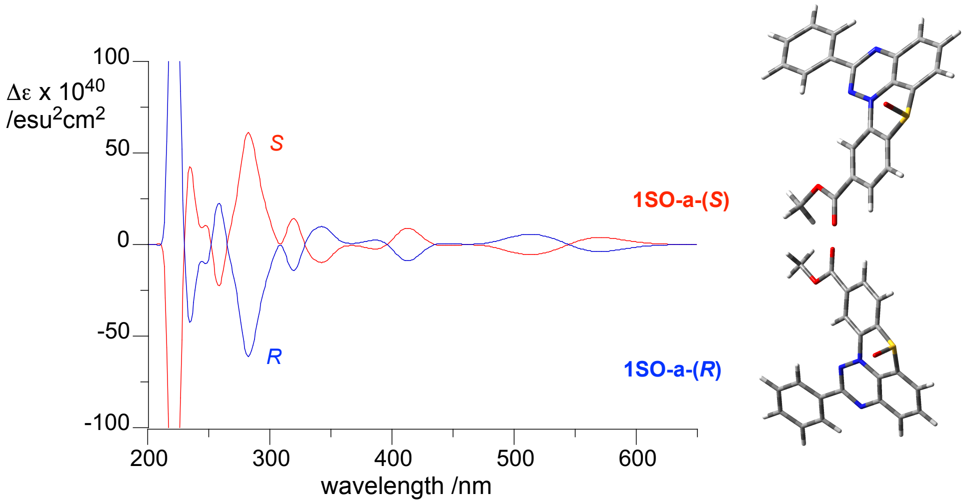

Figure S32. Left: Calculated (DFT) electronic circular dichroism spectra for two enantiomers of **1SO-b** in CH_2Cl_2 dielectric medium. Right: UB3LYP/6-311G(2d,p) optimized structures of the two enantiomers.

g) DFT calculations of sulfoxides racemization

The racemization of radical **1SO-a** and model sulfoxide Tol–SO–Ph was investigated using density functional theory (DFT) at the (U)B3LYP/6-311G(2d,p) level in the gas phase. Geometry optimizations were carried out for both enantiomers and the corresponding transition state (TS). Harmonic frequency calculations confirmed the nature of the stationary points, with no imaginary frequencies for the minima and a single imaginary frequency for the TS, corresponding to inversion at the sulfur stereogenic center. Thermochemical parameters were obtained within the rigid-rotor harmonic-oscillator approximation at 298 K, allowing determination of activation enthalpy ($\Delta H^{\ddagger}$), activation free energy ($\Delta G^{\ddagger}_{298}$), and entropy ($\Delta S^{\ddagger}$) of racemization. The calculated activation barrier supports the experimentally observed high configurational stability of the sulfoxide radical at ambient

temperature. The results are collected in Table S10, while GS and TS geometries for **1SO-a** are shown in Figure S33.

Table S10. Activation parameters for racemization of sulfoxides obtained using the UB3LYP/6-311G(2d,p) method.

sulfoxide	$\Delta H^{\ddagger}$ /kcal mol^{-1}	$\Delta S^{\ddagger}$ /cal mol^{-1}K^{-1}	$\Delta G^{\ddagger}_{298}$ /kcal mol^{-1}
Tolyl–SO–Ph	38.6<br>(36.2) [a]	-2.59<br>(-5.1) [a]	39.4<br>(37.7) [a]
**1SO-a**	36.8	0.31	36.7

[a] Experimental values obtained in xylene. Ref.[12].

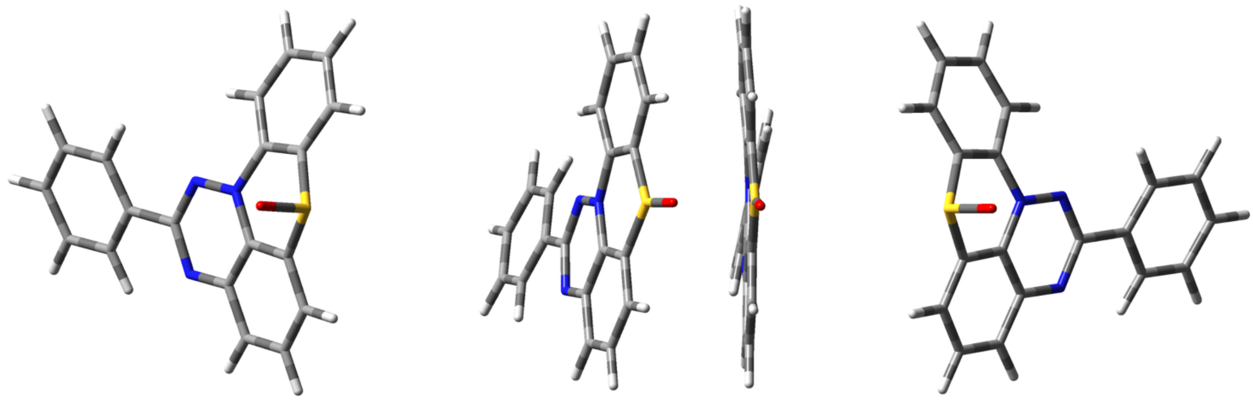

1SO-a-(*R*) **1SO-a-(TS)** **1SO-a-(*S*)**

Figure S33. Geometries of the GS and the TS in racemization of chiral sulfoxide **1SO-a** obtained at the B3LYP/6-311G(2d,p) level of theory in gas phase.

9. Archive for UB3LYP/6-311G(2d,p) geometry optimization results.

1S-a

1\1\GINC-LOCALHOST\FOpt\UB3LYP\6-311G(2d,p)\C19H12N3S1(2)\PK\15-Sep-20
25\0\\#P UB3LYP/6-311G(2d,p) FOpt(tight) SCF=Direct Geom=(NoDistance,N
oAngle) fcheck\\C(8)-S-Ph(N1) benzotrazinyl (phenazinoBT)\\0,2\N,0.582
6482818,-0.2074079911,0.0806253743\N,-1.4271764919,1.6533695325,-0.237
4265585\C,-1.6434935636,0.3580062955,-0.0245110951\N,-0.7188082769,-0.
5920368299,0.1216881195\C,2.5206473574,2.9581599252,0.0044795103\C,2.2
475279967,1.604332085,0.1473702884\C,0.9327226431,1.1438648076,0.01726
41018\C,-0.1269742211,2.0630616364,-0.1909454805\C,0.1793796655,3.4279
791754,-0.3216346191\C,1.4850719516,3.8634283532,-0.2317933379\C,1.521
1386303,-1.2594260783,-0.0634808498\C,2.8956521424,-1.0447619887,0.110
373405\C,3.7862942398,-2.1031304647,-0.0508636032\C,3.3299538551,-3.37
46017128,-0.3640155199\C,1.9673110407,-3.5877553899,-0.5420910448\C,1.

0716166155,-2.5404671553,-0.4025016455\C,-3.0538642839,-0.1071307037,0
.0461129184\C,-3.3691427355,-1.414178513,0.4310832123\C,-4.6923586789,
-1.8279759094,0.493401936\C,-5.7184612991,-0.9456885811,0.1696300819\C
,-5.4124019031,0.3563737012,-0.2122207249\C,-4.0903564502,0.7754096494
,-0.2708254424\S,3.5697052837,0.5177194336,0.6015725746\H,-0.643183306
1,4.1118335371,-0.4832728313\H,4.846705766,-1.9199574972,0.0753117244\
H,0.0138951544,-2.6898692793,-0.5512612473\H,-2.5699617341,-2.09508955
9,0.6895824404\H,-4.9243043229,-2.8419633964,0.7974770534\H,-6.2069016
167,1.0490553748,-0.4639325606\H,-3.8397284562,1.7862584937,-0.5610568
262\H,1.7147966611,4.9165261646,-0.3379840128\H,3.5429023445,3.3058523
796,0.0860920688\H,4.0355705789,-4.1879170761,-0.4777148174\H,-6.75117
23144,-1.2709304169,0.2175769954\H,1.596752446,-4.5718510018,-0.800650
5877\\Version=ES64L-G16RevB.01\State=2-A\HF=-1293.925899\S2=0.765703\S
2-1=0.\S2A=0.750186\RMSD=9.334e-09\RMSF=2.095e-07\Dipole=0.5992303,-0.
3136805,-0.1485933\Quadrupole=3.7762412,6.8810117,-10.6572529,0.513116
9,-0.1318886,-0.1973936\PG=C01 [X(C19H12N3S1)]\\@

1S-b

1\1\GINC-LOCALHOST\FOpt\UB3LYP\6-311G(2d,p)\C21H14N3O2S1(2)\PK\17-Sep-
2025\0\\#P UB3LYP/6-311G(2d,p) FOpt(tight) SCF=Direct Geom=(NoDistance
,NoAngle) fcheck\\C(8)-S-Ph(N1) benzotrazinyl-COOMe\\0,2\N,3.649696696
4,-1.973814664,2.3320536478\N,4.3284771782,-1.912179463,5.0030646892\C
,4.2842590544,-3.0256076578,4.2757876923\N,3.95086339,-3.123407793,2.9
879529472\C,3.7722412377,1.6811824411,2.9731278881\C,3.6675121809,0.48
71181977,2.2731549254\C,3.8030723856,-0.7299320591,2.9490794626\C,4.11
14602417,-0.7444962803,4.333238783\C,4.2202034115,0.4801956109,5.01365
43185\C,4.0457529185,1.6718096518,4.341855754\C,3.0347355698,-2.143520
7148,1.0661023795\C,2.8724526336,-1.0699188391,0.1773785914\C,2.263726
694,-1.2825493952,-1.0594279306\C,1.828748664,-2.5411043214,-1.4294178
383\C,1.979323047,-3.6102436765,-0.5457163877\C,2.5710465537,-3.407201
6525,0.6960495932\C,4.6228980736,-4.3075982933,4.9475792116\C,4.740822
9757,-5.4982702332,4.2232368869\C,5.0611940052,-6.6837992966,4.8695564
619\C,5.2647312496,-6.6990608131,6.2459744092\C,5.1503387963,-5.518392
4204,6.972412233\C,4.8348091609,-4.3296163971,6.3290551666\S,3.4627107
676,0.5596661637,0.51503757\H,4.4526862626,0.4462516503,6.0696566433\H
,2.136074613,-0.4435575821,-1.7327086915\H,2.6817552674,-4.2248612678,
1.3880155299\H,4.5887515885,-5.4828169125,3.1528846097\H,5.1542527231,
-7.5992608737,4.2971445282\H,5.3091110401,-5.5231095061,8.0443685469\H
,4.7476225433,-3.4046282531,6.88186969\H,4.1268825275,2.6120369378,4.8
731538623\H,3.647488813,2.6194587299,2.4471139149\H,1.3626527874,-2.71
42940199,-2.389746913\H,5.5137405337,-7.6261738787,6.7489670031\C,1.48
10985084,-4.9468694886,-0.9739577547\O,0.9575070323,-5.1644506799,-2.0
405160044\O,1.6773213468,-5.8972661855,-0.0382410001\C,1.2129490777,-7
.2095224023,-0.3941582454\H,1.7286085448,-7.5661984462,-1.2854425466\H
,1.4404713172,-7.8409118648,0.4606294995\H,0.1406126872,-7.1936975616,
-0.5870927971\\Version=ES64L-G16RevB.01\State=2-A\HF=-1521.8732816\S2=
0.765878\S2-1=0.\S2A=0.750189\RMSD=5.840e-09\RMSF=1.658e-07\Dipole=0.0
022538,0.2354326,0.2316564\Quadrupole=-9.0847521,15.5068284,-6.4220763
,-0.138039,-1.2443081,-1.9207472\PG=C01 [X(C21H14N3O2S1)]\\@

1SO-a-*R*

1\1\GINC-LOCALHOST\FOpt\UB3LYP\6-311G(2d,p)\C19H12N3O1S1(2)\PK\05-May-
2026\0\\#P UB3LYP/6-311G(2d,p) FOpt(tight) SCF=Direct Geom=(NoDistance
,NoAngle) fcheck # freq(noraman)\\C(8)-SO-Ph(N1) benzotrazinyl (phenaz
inoBT), S-oxide, R config O is axial\\0,2\N,0.3409217932,0.2682188655,
0.0555420965\N,-1.6020177637,-1.6549898824,-0.2560679542\C,-1.86598474
7,-0.3654623282,-0.0374424888\N,-0.9726552439,0.6145667722,0.091282488
3\C,2.3957552663,-2.8070059966,-0.1056650168\C,2.066772761,-1.47161433
2,0.094847589\C,0.7358751026,-1.0593939324,-0.006701868\C,-0.290878250
6,-2.0135619101,-0.232837322\C,0.0761167563,-3.356770647,-0.4312200011
\C,1.4014597616,-3.7445348549,-0.3859425421\C,1.2554810966,1.333274445
6,-0.0758778706\C,2.6348460423,1.1256030726,0.0641422436\C,3.524110088
9,2.1718736026,-0.1656865723\C,3.062159973,3.4295487096,-0.517058669\C
,1.6893886805,3.6407989001,-0.6297288887\C,0.7908475289,2.6097713938,-
0.4179522224\C,-3.2905466056,0.0462024223,0.0497985433\C,-3.6483650738
,1.3357044451,0.4563190523\C,-4.9848164088,1.7004485497,0.5350055621\C
,-5.9810107594,0.7860059925,0.2068939887\C,-5.6323068038,-0.4988743702
,-0.1957632369\C,-4.2967722337,-0.8692602107,-0.2713333797\S,3.3513036
765,-0.3857246678,0.7338628661\H,-0.7192011772,-4.0671230665,-0.615887
6412\H,4.5859127843,1.9885111148,-0.0470308779\H,-0.270578687,2.764885
8838,-0.5319471053\H,-2.8725418024,2.0413156239,0.7196603563\H,-5.2505
705844,2.7005792131,0.8563319231\H,-6.4036462252,-1.2160500639,-0.4503
808096\H,-4.0139606961,-1.8666205667,-0.5780129196\H,1.6695983896,-4.7
81350299,-0.545570658\H,3.431487612,-3.1133246359,-0.0188761652\H,3.75
99360238,4.2382609743,-0.6929706799\H,-7.0242064689,1.0730076796,0.268
3226304\H,1.3124882632,4.6198259091,-0.8997188141\O,3.191506671,-0.341
7138359,2.2198500738\\Version=ES64L-G16RevB.01\State=2-A\HF=-1369.1269
828\S2=0.768967\S2-1=0.\S2A=0.750261\RMSD=2.019e-09\RMSF=1.980e-07\Dip
ole=-0.143825,0.3273159,-1.3468867\Quadrupole=3.3117805,11.4480907,-14
.7598712,-0.0054555,-8.23446,1.1740771\PG=C01 [X(C19H12N3O1S1)]\\@

1SO-a-*S*

1\1\GINC-LOCALHOST\FOpt\UB3LYP\6-311G(2d,p)\C19H12N3O1S1(2)\PK\05-May-
2026\0\\#P UB3LYP/6-311G(2d,p) FOpt(tight) SCF=Direct Geom=(NoDistance
,NoAngle) fcheck # freq(noraman) guess=check\\C(8)-SO-Ph(N1) benzotraz
inyl (phenazinoBT), S-oxide, S config O is axial\\0,2\N,0.405106,-0.25
0043,-0.03519\N,-1.577933,1.638322,-0.304406\C,-1.813767,0.346854,-0.0
65972\N,-0.900866,-0.617277,0.044146\C,2.40302,2.856388,-0.287701\C,2.
102412,1.517834,-0.064953\C,0.77601,1.083135,-0.121701\C,-0.272792,2.0
18308,-0.324833\C,0.065742,3.365313,-0.546693\C,1.385272,3.774929,-0.5
4579\C,1.332657,-1.301353,-0.184963\C,2.71205,-1.070009,-0.089383\C,3.
610996,-2.103896,-0.336906\C,3.15931,-3.372225,-0.662374\C,1.787403,-3
.606775,-0.73079\C,0.878885,-2.588453,-0.500884\C,-3.228001,-0.086988,
0.068848\C,-3.551875,-1.378136,0.497973\C,-4.879088,-1.76372,0.621101\
C,-5.899818,-0.868756,0.31546\C,-5.584859,0.417661,-0.109481\C,-4.2585
59,0.808901,-0.229517\S,3.423658,0.459125,0.544109\H,-0.746471,4.06086
6,-0.713273\H,4.672766,-1.902212,-0.252678\H,-0.182832,-2.761845,-0.58
0722\H,-2.756812,-2.068504,0.74375\H,-5.118338,-2.764879,0.959595\H,-6
.375363,1.119748,-0.346784\H,-4.001768,1.807708,-0.553871\H,1.631252,4
.814367,-0.723069\H,3.435731,3.180278,-0.235647\H,3.864616,-4.17118,-0
.852283\H,-6.935765,-1.172024,0.411599\H,1.418612,-4.594341,-0.980087\
O,3.310212,0.426868,2.034653\\Version=ES64L-G16RevB.01\State=2-A\HF=-1
369.1269828\S2=0.768967\S2-1=0.\S2A=0.750261\RMSD=3.630e-09\RMSF=4.275
e-07\Dipole=-0.1795951,-0.3425751,-1.3387714\Quadrupole=2.7938078,11.4
184594,-14.2122672,-0.2533681,-8.7499302,-1.5529305\PG=C01 [X(C19H12N3
O1S1)]\\@

1SO-b-*R*

1\1\GINC-LOCALHOST\FOpt\UB3LYP\6-311G(2d,p)\C21H14N3O3S1(2)\PK\05-May-
2026\0\\#P UB3LYP/6-311G(2d,p) FOpt(tight) SCF=Direct Geom=(NoDistance

,NoAngle) fcheck\\C(8)-SO-Ph(N1) benzotrazinyl (phenazinoBT), 10-COOMe
, R isomer, axial\\0,2\N,-0.0554062869,-0.6689162404,-0.1621021117\N,2
.3801817856,-1.8178124206,0.3972181611\C,2.2319887059,-0.5466077121,0.
0189582706\N,1.0803963573,0.0732522432,-0.235915292\C,-1.0361056796,-4
.1973714572,0.3808376345\C,-1.1383752049,-2.8607629299,0.0132443838\C,
-0.0123229653,-2.0354564437,0.0662184475\C,1.2530145827,-2.5769848902,
0.4149327184\C,1.3218377022,-3.9333915745,0.7809962998\C,0.190820613,-
4.7269602054,0.7812123464\C,-1.265378929,0.0545868079,-0.1695951252\C,
-2.5008107242,-0.5977270139,-0.2855548033\C,-3.6849720185,0.1295289673
,-0.1898560641\C,-3.6609337177,1.4980235674,0.0010920871\C,-2.42989768
26,2.1541333402,0.0888880807\C,-1.2411718144,1.4417033896,0.0094108442
\C,3.4546818963,0.2859196156,-0.1131740789\C,3.4005389181,1.569717384,
-0.6656792653\C,4.5547335061,2.3306637902,-0.7840564647\C,5.775948573,
1.8235178649,-0.350756854\C,5.8371407774,0.5468370949,0.1978285666\C,4
.685875321,-0.2196972505,0.3138283634\S,-2.669428885,-2.3279263392,-0.
76596963\H,2.2922385421,-4.32494293,1.0570438757\H,-4.6288777406,-0.39
42979424,-0.2859815753\H,-0.2939450944,1.9456021859,0.1046099317\H,2.4
518892477,1.9585337942,-1.0091358297\H,4.5022042687,3.3218583952,-1.21
86688517\H,6.7855297263,0.1457610869,0.5351293547\H,4.72213982,-1.2150
61635,0.7341326812\H,0.2582450352,-5.7681707402,1.0703607473\H,-1.9164
815643,-4.827005538,0.3301653773\H,-4.5709712004,2.0771163947,0.075274
9288\H,6.6760677421,2.4198024368,-0.4435691063\C,-2.445101125,3.635134
1052,0.2888022269\O,-3.45381379,4.290441719,0.3855099557\O,-1.20796321
44,4.1615081773,0.3494269581\C,-1.1580488213,5.5856833749,0.5417898135
\H,-0.1016480845,5.8384081463,0.5711662941\H,-1.6548584999,6.096546901
4,-0.2824175509\H,-1.6467084812,5.8590247721,1.4764622297\O,-2.4612086
465,-2.3989106919,-2.2444853957\\Version=ES64L-G16RevB.01\State=2-A\HF
=-1597.072812\S2=0.76937\S2-1=0.\S2A=0.750274\RMSD=2.212e-09\RMSF=3.61
8e-07\Dipole=0.7308381,0.2302519,1.2599405\Quadrupole=-0.7407315,12.64
88838,-11.9081523,6.0540154,-4.2926387,-8.6066918\PG=C01 [X(C21H14N3O3
S1)]\\@

1SO-b-*S*

1\1\GINC-LOCALHOST\FOpt\UB3LYP\6-311G(2d,p)\C21H14N3O3S1(2)\PK\05-May-
2026\0\\#P UB3LYP/6-311G(2d,p) FOpt(tight) SCF=Direct Geom=(NoDistance
,NoAngle) fcheck\\C(8)-SO-Ph(N1) benzotrazinyl (phenazinoBT), 10-COOMe
, S isomer, axial\\0,2\N,-0.0554087155,-0.6689159347,0.1621029539\N,2.
3801747272,-1.8178218202,-0.397217642\C,2.2319867495,-0.5466164967,-0.
0189577505\N,1.0803969098,0.0732480128,0.2359159618\C,-1.0361222185,-4
.1973672399,-0.3808366424\C,-1.138386386,-2.8607582919,-0.0132434183\C
,-0.0123308563,-2.0354563053,-0.0662175765\C,1.2530045018,-2.576989790
4,-0.4149319836\C,1.321822184,-3.9333967818,-0.7809954994\C,0.19080192
9,-4.7269609048,-0.781211409\C,-1.2653784773,0.0545919411,0.1695962493
\C,-2.5008128482,-0.5977169539,0.2855561334\C,-3.6849712638,0.12954377
03,0.1898577584\C,-3.6609275504,1.4980382919,-0.0010902227\C,-2.429888
9147,2.1541431521,-0.0888864807\C,-1.2411658761,1.4417084421,-0.009409
6087\C,3.4546832777,0.28590597,0.1131743242\C,3.4005454142,1.569704246
1,0.6656788378\C,4.5547430609,2.3306460358,0.7840558305\C,5.7759561497
,1.8234949367,0.3507566987\C,5.8371432723,0.5468136364,-0.1978280539\C
,4.6858747245,-0.2197160984,-0.3138276626\S,-2.6694379207,-2.327915671
6,0.7659706585\H,2.2922214387,-4.3249520111,-1.0570431768\H,-4.6288790
53,-0.3942793871,0.2859834395\H,-0.2939371843,1.945603469,-0.104608908
4\H,2.4518972698,1.9585246765,1.0091350511\H,4.5022177611,3.3218410826
,1.2186676792\H,6.7855306565,0.1457336021,-0.5351284636\H,4.7221352573
,-1.215080843,-0.7341314744\H,0.258222175,-5.7681717346,-1.0703597289\
H,-1.9165006176,-4.8269978011,-0.3301643099\H,-4.5709627379,2.07713476
77,-0.0752727647\H,6.6760777214,2.4197759066,0.4435688053\C,-2.4450864
892,3.6351439904,-0.288800535\O,-3.453796559,4.2904556095,-0.385508183
\O,-1.207946497,4.161513173,-0.3494252013\C,-1.1580264801,5.5856881956
,-0.5417879058\H,-0.1016247518,5.838408927,-0.5711634362\H,-1.64668423

24,5.8590315179,-1.476460762\H,-1.6548349632,6.0965535719,0.2824190242
\O,-2.4612180277,-2.3989011184,2.2444864145\\Version=ES64L-G16RevB.01\
State=2-A\HF=-1597.072812\S2=0.76937\S2-1=0.\S2A=0.750274\RMSD=2.184e-
09\RMSF=3.610e-07\Dipole=0.730839,0.2302492,-1.2599403\Quadrupole=-0.7
406823,12.6488371,-11.9081548,6.0540666,4.2926727,8.6066744\PG=C01 [X(
C21H14N3O3S1)]\\@

1SO_2-a

1\1\GINC-LOCALHOST\FOpt\UB3LYP\6-311G(2d,p)\C19H12N3O2S1(2)\PK\16-Sep-
2025\0\\#P UB3LYP/6-311G(2d,p) FOpt(tight) SCF=Direct Geom=(NoDistance
,NoAngle) fcheck\\C(8)-SO2-Ph(N1) benzotrazinyl (phenazinoBT), SO2\\0,
2\N,-0.3488500654,0.2644442402,0.0864053347\N,1.5953746732,-1.65620606
26,-0.2514987345\C,1.859755114,-0.3674620641,-0.0274187637\N,0.9656381
598,0.6104834098,0.1139703122\C,-2.4018215271,-2.8278086187,-0.0615848
61\C,-2.0636363878,-1.4970369041,0.1421886745\C,-0.739390492,-1.067752
6717,0.0254616868\C,0.2863223762,-2.019115726,-0.2149125174\C,-0.07941
06969,-3.36354845,-0.4072579027\C,-1.4026691408,-3.7575188034,-0.34815
28872\C,-1.2585587409,1.3375092934,-0.050075294\C,-2.6398556711,1.1515
84286,0.1006291713\C,-3.5324468913,2.1911656124,-0.1363354526\C,-3.058
8434927,3.4420531691,-0.4938851461\C,-1.6850994224,3.6427782211,-0.618
2151462\C,-0.7906422082,2.6077399586,-0.4095524066\C,3.2838619237,0.04
57590165,0.0512407316\C,3.6428138328,1.332465661,0.4657890467\C,4.9792
346227,1.6987012844,0.5362868935\C,5.9739396049,0.7886743345,0.1917553
241\C,5.6240706419,-0.4933016741,-0.2190754195\C,4.2886447629,-0.86542
16225,-0.2864126063\S,-3.2915078941,-0.3697534469,0.734165499\H,0.7163
954929,-4.0707294009,-0.6013928043\H,-4.5924908681,1.9994937231,-0.030
9393956\H,0.2701881752,2.7539837007,-0.5376244528\H,2.8684339255,2.034
8458587,0.7417771722\H,5.2461858284,2.6964141545,0.8639104484\H,6.3943
808424,-1.206776826,-0.486634462\H,4.0053323055,-1.8605941655,-0.59959
81909\H,-1.6676002616,-4.7948116409,-0.5088805326\H,-3.4399299358,-3.1
232342184,0.0148439184\H,-3.751692117,4.2537819326,-0.6742872006\H,7.0
171123029,1.0769178727,0.2466787371\H,-1.3042444727,4.616849437,-0.899
8206734\O,-4.5684379912,-0.6552594617,0.1093777358\O,-3.2018420081,-0.
3307609285,2.1821224831\\Version=ES64L-G16RevB.01\State=2-A\HF=-1444.3
724353\S2=0.769405\S2-1=0.\S2A=0.750273\RMSD=4.222e-09\RMSF=3.551e-07\
Dipole=1.0707115,0.515347,-1.0107966\Quadrupole=-4.8952655,15.7747516,
-10.8794861,-2.2960433,5.2648297,0.690351\PG=C01 [X(C19H12N3O2S1)]\\@

1SO_2-b

1\1\GINC-LOCALHOST\FOpt\UB3LYP\6-311G(2d,p)\C21H14N3O4S1(2)\PK\17-Sep-
2025\0\\#P UB3LYP/6-311G(2d,p) FOpt(tight) SCF=Direct Geom=(NoDistance
,NoAngle) fcheck\\C(8)-SO2-Ph(N1) benzotrazinyl-COOMe\\0,2\N,-0.192144
136,0.2889899818,0.0084720599\N,1.7499467062,-1.643459383,-0.258321880
7\C,2.0149399547,-0.3572521783,-0.0196505583\N,1.1223153631,0.62630568
73,0.0892363184\C,-2.2595842591,-2.7879115796,-0.2349327066\C,-1.92062
19518,-1.4603106513,-0.0116053575\C,-0.5897500272,-1.0397236168,-0.073
0022965\C,0.4383963485,-1.9973545475,-0.275724167\C,0.0713135217,-3.33
84506966,-0.488337642\C,-1.2559103693,-3.7233011224,-0.4847185434\C,-1
.0886129237,1.3686720983,-0.1584597243\C,-2.476133918,1.193124084,-0.0
628747329\C,-3.3507424208,2.241578135,-0.3300635011\C,-2.8552964462,3.
488019304,-0.6609155475\C,-1.4726800285,3.6829246156,-0.7306013971\C,-
0.5953865621,2.6343499447,-0.4914057701\C,3.4373385896,0.0454623616,0.
1173899765\C,3.7883497213,1.333026957,0.5363354246\C,5.1234824154,1.68
86456494,0.6626788863\C,6.1246135048,0.7674916602,0.3701749288\C,5.782
4164754,-0.5150600346,-0.0452521796\C,4.4481651691,-0.8767819545,-0.16
84418734\S,-3.1636930092,-0.3283628466,0.5360812939\H,0.8693506181,-4.
0505040565,-0.6531975655\H,-4.4158013822,2.059788814,-0.2684404837\H,0
.4682718753,2.7800848147,-0.5762294354\H,3.0089249759,2.0447596058,0.7
709261679\H,5.3843400261,2.6869455333,0.9933853888\H,6.5576557215,-1.2
370532863,-0.2726006257\H,4.1706449234,-1.8722005164,-0.4859310972\H,-

1.5212329083,-4.7580106593,-0.6604840489\H,-3.3018416126,-3.0766194857
,-0.2018184217\H,-3.5133655324,4.3207174478,-0.866966635\H,7.166768070
5,1.0476002959,0.4689367396\O,-4.4155429983,-0.6008477494,-0.141459176
4\O,-3.1305625595,-0.2895786429,1.9860149816\C,-0.9815680954,5.0480794
522,-1.0925413818\O,-1.7079157856,5.9825351737,-1.3261738556\O,0.36128
48189,5.1209313219,-1.1284514098\C,0.8949603006,6.4117508555,-1.472470
2558\H,1.9748782639,6.2925583015,-1.4600226998\H,0.5506571844,6.712033
0934,-2.4615229043\H,0.5797362679,7.1575998691,-0.7435437119\\Version=
ES64L-G16RevB.01\State=2-A\HF=-1672.3178886\S2=0.769779\S2-1=0.\S2A=0.
750285\RMSD=4.113e-09\RMSF=1.364e-07\Dipole=1.7358852,0.0331325,-0.856
464\Quadrupole=-3.7324112,12.6199446,-8.8875334,5.7684757,3.4997266,3.
2459284\PG=C01 [X(C21H14N3O4S1)]\\@

10. References

(1) Bartos, P.; Celeda, M.; Pietrzak, A.; Kaszyński, P. Planar Blatter radicals through Bu_3SnH- and TMS_3SiH-assisted cyclization of aryl iodides: azaphilic radical addition. *Org. Chem. Front.* **2022**, *9*, 929–938.

(2) *CrysAlis PRO, Agilent Technologies Ltd, Yarnton, Oxfordshire, England*; 2014.

(3) Sheldrick, G. SHELXT: Integrated Space-Group and Crystal-Structure Determination. *Acta Crystallogr. A* **2015**, *71*, 3–8.

(4) Sheldrick, G. Crystal Structure Refinement with SHELXL. *Acta Crystallogr. B* **2015**, *71* (1), 3-8.

(5) Connelly, N. G.; Geiger, W. E. Chemical Redox Agents for Organometallic Chemistry. *Chem. Rev.* **1996**, *96*, 877–910.

(6) Hansch, C.; Leo, A.; Taft, R. W. A Survey of Hammett Substituent Constants and Resonance and Field Parameters. *Chem. Rev.* **1991**, *91*, 165–195.

(7) *Gaussian 16 Rev. C.01*; Wallingford, CT, 2016.

(8) Cossi, M.; Scalmani, G.; Rega, N.; Barone, V. New Developments in the Polarizable Continuum Model for Quantum Mechanical and Classical Calculations on Molecules in Solution. *J. Chem. Phys.* **2002**, *117*, 43–54.

(9) De Vleeschouwer, F.; Chankisjijev, A.; Yang, W.; Geerlings, P.; De Proft, F. Pushing the Boundaries of Intrinsically Stable Radicals: Inverse Design Using the Thiadiazinyl Radical as a Template. *J. Org. Chem.* **2013**, *78*, 3151–3158.

(10) Yamaguchi, K.; Takahara, Y.; Fueno, T.; Nasu, K. Ab initio MO Calculations of Effective Exchange Integrals Between Transition-Metal Ions via Oxygen Dianions: Nature of the Copper-Oxygen Bonds and Superconductivity. *Jpn. J. Appl. Phys.* **1987**, *26*, L1362.

(11) Stratmann, R. E.; Scuseria, G. E.; Frisch, M. J. An Efficient Implementation of Time-dependent Density-functional Theory for the Calculation of Excitation Energies of Large Molecules. *J. Chem. Phys.* **1998**, *109*, 8218–8224.

(12) Rayner, D. R.; Gordon, A. J.; Mislow, K. Thermal Racemization of Diaryl, Alkyl Aryl, and Dialkyl Sulfoxides by Pyramidal Inversion. *J. Am. Chem. Soc.* **1968**, *90*, 4854–4860.
